# Analysis of Wnt signaling β-catenin spatial dynamics in HEK293T cells

**DOI:** 10.1186/1752-0509-8-44

**Published:** 2014-04-08

**Authors:** Chin Wee Tan, Bruce S Gardiner, Yumiko Hirokawa, David W Smith, Antony W Burgess

**Affiliations:** 1 Structural Biology Division, The Walter and Eliza Hall Institute of Medical Research, Parkville, VIC, Australia; 2 Department of Medical Biology, University of Melbourne, Parkville, VIC, Australia; 3 Ludwig Institute for Cancer Research, Melbourne-Parkville Branch, Parkville, VIC, Australia; 4 Department of Infrastructure Engineering, University of Melbourne, Parkville, VIC, Australia; 5 School of Computer Science and Software Engineering, The University of Western Australia, Perth, WA, Australia; 6 Department of Surgery, University of Melbourne, Parkville, VIC, Australia

**Keywords:** Wnt signaling, HEK293T, β-catenin, Computational model, Compartmentalization, Confocal microscopy, Systems biology

## Abstract

**Background:**

Wnt/β-catenin signaling is involved in different stages of mammalian development and implicated in various cancers (e.g. colorectal cancer). Recent experimental and computational studies have revealed characteristics of the pathway, however a cell-specific spatial perspective is lacking. In this study, a novel 3D confocal quantitation protocol is developed to acquire spatial (two cellular compartments: nucleus and cytosol-membrane) and temporal quantitative data on target protein (e.g. β-catenin) concentrations in Human Epithelial Kidney cells (HEK293T) during perturbation (with either cycloheximide or Wnt3A). Computational models of the Wnt pathway are constructed and interrogated based on this data.

**Results:**

A single compartment Wnt pathway model is compared with a simple β-catenin two compartment model to investigate Wnt3A signaling in HEK293T cells. When protein synthesis is inhibited, β-catenin decreases at the same rate in both cellular compartments, suggesting diffusional transport is fast compared to β-catenin degradation in the cytosol. With Wnt3A stimulation, the total amount of β-catenin rises throughout the cell, however the increase is initially (~first hour) faster in the nuclear compartment. While both models were able to reproduce the whole cell changes in β-catenin, only the compartment model reproduced the Wnt3A induced changes in β-catenin distribution and it was also the best fit for the data obtained when active transport was included alongside passive diffusion transport.

**Conclusions:**

This integrated 3D quantitation imaging protocol and computational modeling approach allowed cell-specific compartment models of the signaling pathways to be constructed and analyzed. The Wnt models constructed in this study are the first for HEK293T and have suggested potential roles of inter-compartment transport to the dynamics of signaling.

## Background

β-catenin is a multi-functional protein involved in cell adhesion, cell migration and gene transcription associated with cell survival, proliferation and differentiation [1-3]. The primary function of the Wnt/β-catenin pathway appears to be the regulation of the β-catenin concentration within the cell [4,5]. It has been proposed previously that in the absence of Wnt signaling, β-catenin forms a complex with the scaffold proteins Axin and Adenomatous Polyposis Coli (APC, itself a multi-functional protein) to form the so-called degradation complex [6,7]. The degradation complex facilitates the phosphorylation of β-catenin by glycogen synthase kinase-3-β (GSK3β) [8], targeting phosphorylated β-catenin for degradation via the proteasome pathway [9]. Activation of the Wnt/β-catenin pathway by Wnt ligands brings about the disruption of the degradation complex and a consequent reduction in β-catenin degradation within the cytoplasm [10,11]. Increased β-catenin concentrations in the cell lead to the formation of the β-catenin: T-Cell Factor complex (TCF, transcription co-factor) in the cell nucleus. The β-catenin-TCF complex activates gene transcription promoting cellular functions which include cell proliferation, survival and cell fate decisions [2,12,13]. However, this general description of the Wnt pathway typically does not take into account the compartment concentrations of proteins, crosstalk with other signaling pathways (e.g. with the cell-cell adhesion pathway) or modulation of pathway behavior due to sequestration to binding partners. Further, this Wnt pathway description has recently been challenged by evidence of APC-independent β-catenin degradation [14,15], APC-associated phosphorylation of β-catenin independent of Axin (and surprisingly mediated by β-catenin itself) [16], Wnt activation of different phospho-forms of β-catenin [17,18] and β-catenin ubiquitination [19]. Clearly the regulation of β-catenin is more complex than the traditional account of Wnt Signaling, so much so that the pathways controlling cytosolic/membrane β-catenin concentrations need to be revisited.

The computational-experimental model developed by Lee and colleagues [20] is still arguably the leading systematic study of Wnt signaling. They studied β-catenin signaling in Xenopus oocyte cytosolic extracts and developed a single compartment computational model to interpret their experimental system under a range of perturbations. This system has many advantages, the foremost of which is the simplicity introduced by focusing on a single homogeneous compartment i.e. the removal of spatial dependencies. In general, more recent models of the Wnt signaling pathway have been based on the Lee model [21-24], as discussed in recent reviews [25,26]. However, to understand the Wnt signaling pathway for human cells, it is logical to want to progress the existing Xenopus Wnt signaling model to one which is parameterized for a mammalian cell system [27]. Even better, to the development of an experimental-computational model of an intact mammalian cell system. However there is only limited mammalian data on the protein concentrations for Wnt signaling components. To begin to overcome data limitations, we recently measured the concentrations of several key Wnt signaling pathway proteins, namely β-catenin, Axin, APC and GSK3β in five mammalian cell-lines [27]. Our results show significant differences between the Xenopus extracts and the protein concentration in mammalian cells. For example in Xenopus, Axin was found to be at low concentrations relative to other pathway proteins, whereas in the mammalian cell lines, it was APC that was at low concentrations. Indeed we have measured the rate of β-catenin degradation in mammalian cell-free extracts (see Additional file 1: Figure S1) and our results indicate that the degradation of mammalian β-catenin is much slower than in the Xenopus oocyte cell-free extracts [20]. These issues highlight the need for caution when extrapolating pathway behavior between cell systems.

Recent Wnt signaling models have progressed towards spatial multi-compartmental models [25,28-31] taking into account spatial complexity of cellular signaling [32]. Schmitz et al. [29,30] developed ODE models, based on a two compartment (nucleus and cytoplasm) model, to study the influence of nucleo-cytoplasmic shuttling of APC and β-catenin on Wnt signaling [29]. Subsequently they extended the model [30] to investigate the impact of nucleo-cytoplasmic shuttling of β-catenin with its component binding partners in the degradation complex. Another study by van Leeuwen et al. [28] in 2007 developed an ODE model of Wnt signaling incorporating different β-catenin pools and three functional ‘compartments’ (i.e. nucleus, cytoplasm and membrane). Another approach to model cellular compartments was taken by Basan and co-workers [31] and involved using a reaction–diffusion model of membrane and cytoplasmic compartments to investigate roles of cytoplasmic β-catenin and α-catenin on cell adhesion and contact inhibition. What was clear from these studies was the need for more quantitative spatial data. The current limitation to multi-scale and multi-compartment modelling is the lack of quantitative spatial and temporal data on the key signaling proteins and complexes (this was also the case for van Leeuwen et al.) [25]. Clearly we need more quantitative data on the Wnt Pathway in mammalian cell systems [27], particularly quantitative spatial temporal data. High-resolution quantitative microscopy techniques enable cellular compartments to be distinguished and quantified, presenting a valuable opportunity for capturing dynamic spatial data of key signaling proteins [25].

The aim of this study was to interrogate the Wnt pathway in HEK293T cells (a human kidney epithelial cell line [33]). HEK293T cells were used in this study, as these cells respond to Wnt3A stimulation (the only cells to do so in the panel of cell lines analyzed in our previous quantitative study [27]) and have no known mutations in the proteins associated with Wnt signaling (therefore providing a comparatively complete system for studying the Wnt signaling mechanism). Specifically we examined the dynamics of the Wnt signaling pathway in structurally intact mammalian HEK293T cells by quantifying changes in the spatial concentration of β-catenin as a result of Wnt3A stimulation or in the presence of cycloheximide. To achieve this aim, a novel 3D confocal quantitative imaging technique was developed. Two theoretical models of the Wnt signaling pathway in HEK293T were constructed with different structures and compartment compositions. The first model is an extension to the single compartment Lee et al. model [20], to include HEK293T data and additional interactions between the key pathway components, and the second model is a simple two compartment model of β-catenin dynamics in the nucleus and cytosol-membrane compartments. A two compartment model was developed to simulate our quantitative compartment data (nuclei and cytosol-membrane). This two-compartment model is structurally similar to the theoretical model published by Schmitz et al. [30] with key differences in the transport mechanisms and data used (see Discussions). Current limitations in image processing methods and membrane markers limit the advancement to a three compartment model. In time, advances in image processing techniques and fluorescent markers will overcome this limitation.

## Results

The 3D confocal microscopy protocol employed in this study was adapted from the confocal compartment cell volume measurement protocol established previously [27]. Specifically, the technique was extended to analyze intact adherent cell cultures stained with fluorescent markers to specifically identify the nuclei region with DAPI and the cell with N-cadherin antibodies. The temporal and spatial perturbations of β-catenin following application of the Wnt pathway activator (Wnt3A ligand) or an inhibitor of protein biosynthesis (cycloheximide) were examined quantitatively. To standardize the intensity levels between samples and time points, 0.3% rated InSpeck™ microspheres were selected as intensity calibration standards, so enabling quantitative microscopy. The selection of microspheres was based on flow cytometry (FACS Calibur) and confocal microscopy analysis (see Additional file 1: Text, Figure S3 and Table S1 for details). This analysis also confirmed that the InSpeck™ intensity microspheres have a stable and specific fluorescent intensity.

### 3D confocal image acquisition of HEK293T

**Figure 1:**
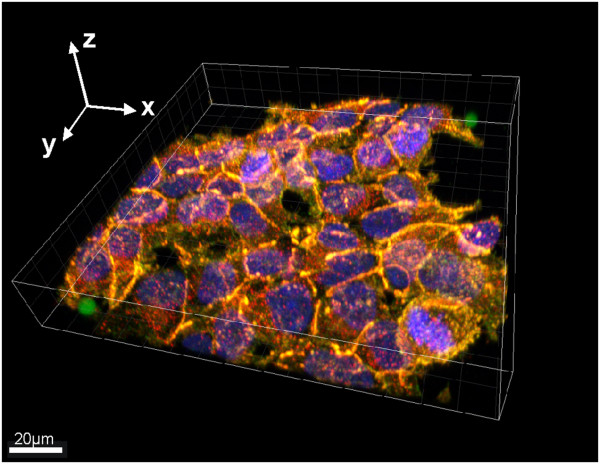
**3D view of a typical acquired image stack for HEK293T cells.** An angled 3D volumetric view with microspheres in green isolated at both corners. Overlayed fluorescent stains: DAPI (nucleus, blue), β-catenin (green) and N-cadherin (red). Co-localization of β-catenin and N-cadherin at cell junctions observed as strong yellow (green + red) fluorescent bands. The image was generated with Imaris x64 Version 7.0.0 (Bitplane AG).

**Figure 2:**
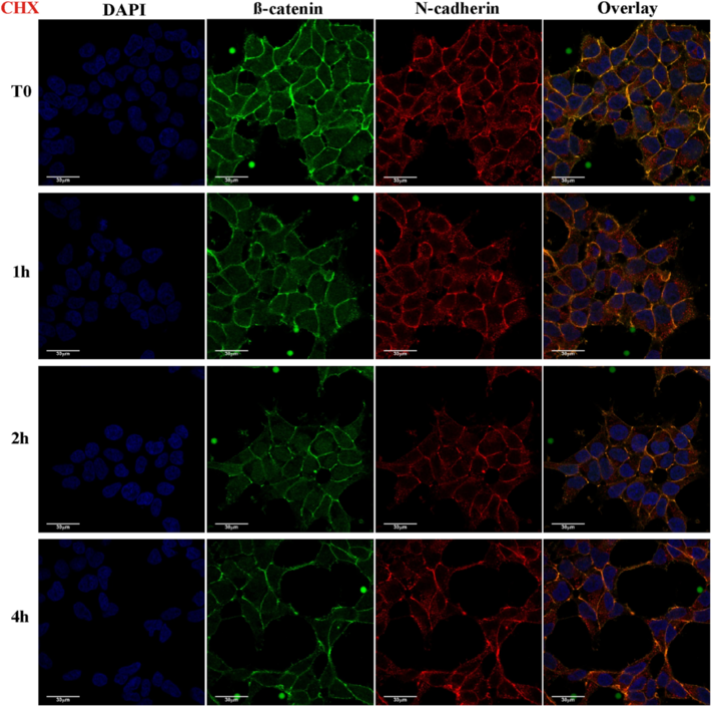
**2D confocal images of CHX inhibited HEK293T cells.** HEK293T cells stimulated for 0, 1, 2 or 4 hours with CHX; showing DAPI (blue), β-catenin (green), N-cadherin (red) and 3 channel overlay. One (of three) independently conducted confocal quantification experiments is shown.

**Figure 3:**
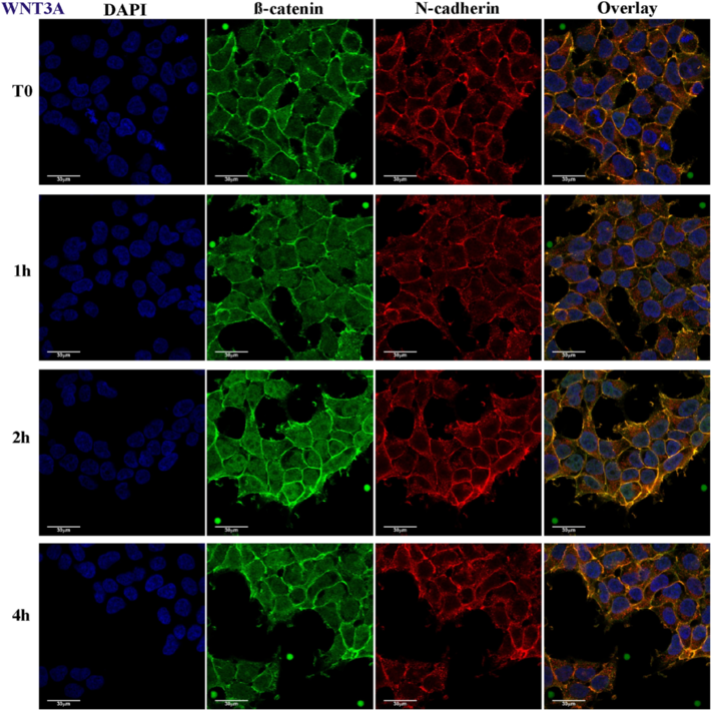
**2D confocal images of Wnt3A stimulated HEK293T cells.** HEK293T cells stimulated for 0, 1, 2 or 4 hours with Wnt3A CM; showing DAPI (blue), β-catenin (green), N-cadherin (red) and 3 channel overlay. One (of three) independently conducted confocal quantification experiments is shown.

Three sets of independent experiments were performed for the two types of perturbation (Wnt3A or CHX), each experiment having 4 time points (0, 1, 2 or 4 hours). Figure 1 shows the 3D volume view of a typical image stack with overlaying fluorescent channels for the nucleus in blue (DAPI), β-catenin in green and N-cadherin in red (cell boundary), together with isolated InSpeck™ microspheres. Figures 2 and 3 show 2D sectional images of the image stack revealing the effects of CHX inhibition and Wnt3A stimulation on β-catenin levels with time.

**Figure 4:**
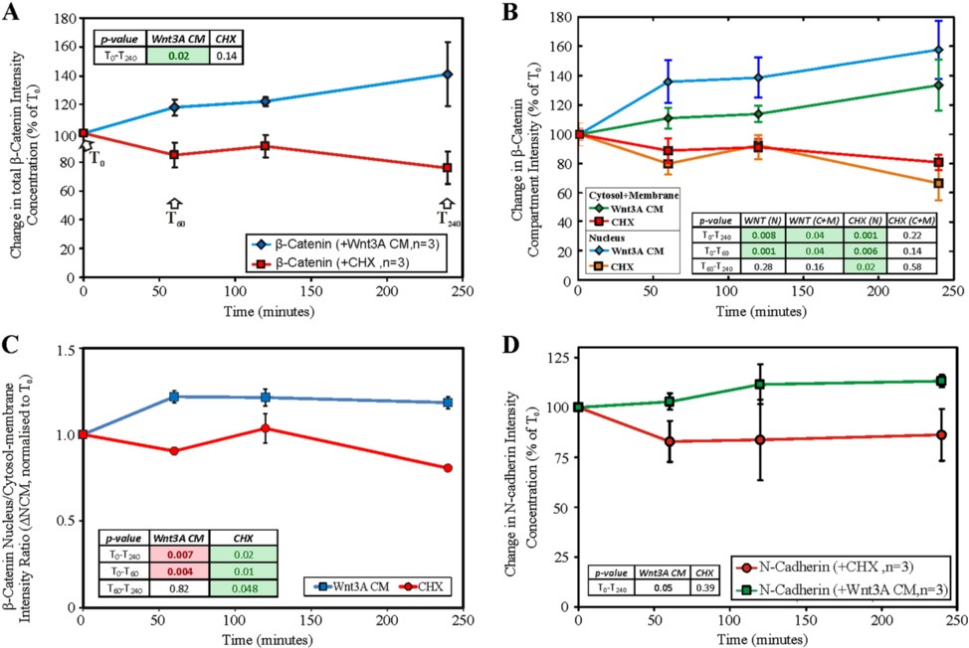
**Quantification of β-catenin and N-cadherin in stimulated/inhibited HEK293T cells.** HEK293T cells were stimulated with Wnt3A CM or inhibited by CHX for 0, 1, 2, or 4 hours. **(A)** Relative change in β-catenin concentration intensity (in terms of % of T_0_); **(B)** Relative change in β-catenin compartment concentration intensity (in terms of % of T_0_); **(C)** β-catenin Nucleus/Cytosol-membrane intensity ratio (normalized to T_0_); **(D)** Relative change in N-cadherin concentration intensity (in terms of % of T_0_); Error Bars: (A and D) Standard deviations, (B and C) Standard Error of the Mean. n = 3; each data point has ≥ 3 image sets. A single factor analysis of variance (ANOVA) test was applied to test for differences between data points for each perturbation, specifically between T_0_-T_240_, T_0_-T_60_ and T_60_-T_240_.

Qualitatively, the 2D images (Figure 2) suggested that endogenous β-catenin levels decreased with time under the influence of CHX, whereas the total endogenous β-catenin levels increased under the influence of Wnt3A (Figure 3). Visual inspection of a representative 2D image would be misleading if it is used to determine the change in β-catenin in different regions of the cell (nucleus, cytosol and membrane) for each time point. Similarly, the total β-catenin of a 3D image stack could not be accurately inferred from a single 2D image. Combining intensities from all 2D images comprising the 3D signals (entire stack of images) gave a clearer estimation. Therefore, the extent of changes in β-catenin levels in the different compartments (nucleus and cytosol-membrane) were quantified using computational and image processing techniques (see Additional file 1: Text, Figures S4 and S5 for details) and the results of the analysis are shown in Figure 4. The quantitative results were consistent with the visual observations. The total concentration of β-catenin in the whole cell for Wnt3A treatment increased with time (45 ± 22% of t_0_ in 4 hours, p-value,T_0_-T_240_ p = 0.02) whereas during CHX treatment β-catenin levels decreased (26 ± 15% of t_0_ in 4 hours, p-value,T_0_-T_240_ p = 0.14). Under Wnt3A stimulation (Figure 4B), the β-catenin level increased in both the nucleus and the cytosol-membrane compartments, however, during the first hour of incubation, the rate of increase in the nuclear compartment was greater than that of the cytosol-membrane compartment (p-value,T_0_-T_60_ N: p < 0.01, CM: p = 0.04). After this initial rapid rise of nuclear β-catenin in the nuclear compartment, the rate of increase in the nuclear compartment concentration of β-catenin reduced (p-value, T_60_-T_240_ N: p = 0.28) and appeared to be similar to the rate of change in the concentration in the cytosol-membrane compartment (p-value, T_60_-T_240_ CM: p = 0.16). For CHX inhibition, a decrease in β-catenin levels was observed in the nuclear compartment (p-value, T_0_-T_240_ N: p < 0.01), with potentially an oscillation observed. For the cytosolic-membrane compartment, the decrease in β-catenin was non-significant during the duration of the experiment (p-value, T_0_-T_240_ CM: p = 0.22).

This redistribution of β-catenin between compartments was quantified using the ratio of the β-catenin level in the nucleus (N) compartment and cytosol-membrane (CM) compartment (the N:CM ratio). An N:CM ratio > 1.0 implies greater nucleus β-catenin concentration than that in the cytosol-membrane compartment. In Figure 4C, the normalized N:CM with respect to T_0_ (∆NCM) was plotted, indicating the shift in β-catenin between the compartments due to the specific perturbations in time (i.e. a greater ∆NCM implies a shift of β-catenin levels to the nuclei compartment as compared to T_0_). Wnt3A stimulation increased the ∆NCM by 0.22 ± 0.04 (p-value,T_0_-T_60_ p < 0.01) within the first hour and was followed by a steady rate of β-catenin increment in both compartments as indicated by a constant ∆NCM of approximately 1.2 (p-value,T_60_-T_240_ p = 0.82). For CHX inhibition, the ∆NCM decreased over the 4 hours of CHX incubation (p-value,T_0_-T_240_ p = 0.02), indicating a gradual shift of β-catenin to the cytosol-membrane compartment. At the onset of Wnt3A stimulation the analyses showed a rapid increase of β-catenin concentration in the nucleus and with CHX inhibition, a slow gradual decrease of β-catenin in both compartments. These observations demonstrated the ability of the protocol to successfully quantify and monitor (in 3D) the concentration of a specific target protein and its localization in intracellular compartments.

Visual inspection of 2D images (Figures 2 and 3) for endogenous N-cadherin in HEK293T cells, in the presence of either Wnt3A or CHX, showed no change with time (up to 4 hours). β-catenin and N-cadherin appeared to be co-located near the membrane, as seen from the yellow overlapping (green and red) signal predominantly at the cell surface. Quantitation of the concentration of total N-cadherin was investigated (Figure 4D). For total N-cadherin, results indicated that Wnt3A stimulation led to an increase of 13 ± 3% after 4 hours (p-value,T_0_-T_240_ p = 0.05). In the presence of CHX, total N-cadherin decreased 17 ± 10% after 1 hour and then remained stable (±1.7%) over the remaining 4 hours (p-value,T_0_-T_240_ p = 0.39).

### Comparison of 3D microscopy quantitation with western blot quantitation

**Figure 5:**
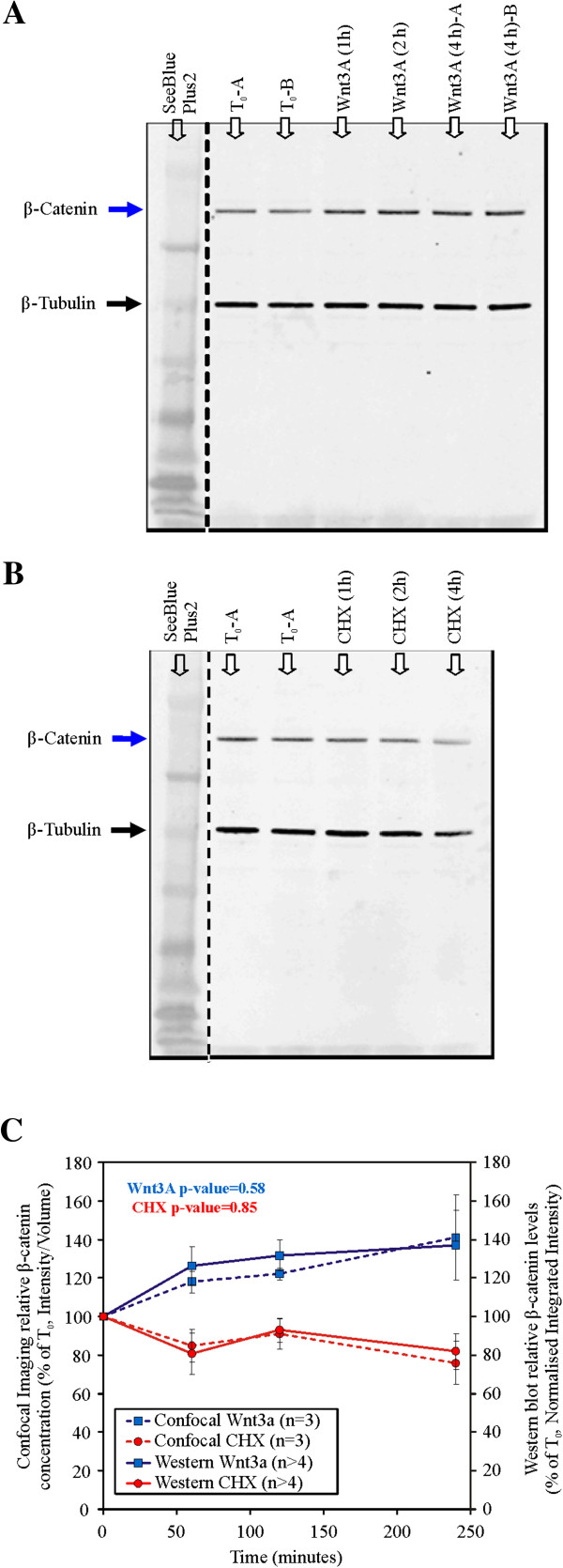
**Confocal 3D quantitation technique validation with quantitative Western blot for HEK293T Whole Cell Lysates.** Western blots for β-catenin:**(A)** Wnt3A stimulation; **(B)** CHX inhibition for 0, 1, 2 or 4 hours; **(C)** Comparison between the results of confocal quantification and Western blot validation. P-values for both Wnt3A and CHX are greater than p = 0.05. (Error Bars: Standard deviation. Selected blots showing non-continuous lanes, replicates are labeled as A or B with β-tubulin used as a loading control.)

To validate the results of the above 3D quantification technique, quantitative Western blots were performed under the same perturbation and culture conditions (as described in the Methods section). Five sets of independent quantitative Western blot experiments were conducted with HEK293T cells. β-tubulin was used as the loading control in these experiments and as a reference for normalization between the samples. Extracts from cells incubated with either Wnt3A or CHX for 0, 1, 2 or 4 hours were loaded onto the gels as shown in Figure 5. Each membrane was immunoblotted for β-catenin and β-tubulin. Upon visual inspection, it could be seen that qualitatively, the levels of total β-catenin increased with Wnt3A incubation and decreased with CHX incubation. The intensities for each band were quantified and normalized. Results of these independent experiments were compared with results obtained using the confocal 3D quantification (as shown in Figure 5C). The two sets of results, that is, the Western blots and the 3D confocal imaging technique, were consistent. This independent validation experiment demonstrated that the 3D confocal quantification technique developed in this study can be used to accurately and reproducibly quantify and monitor the levels of target proteins (in this case β-catenin) in HEK293T cells at different time after stimulation. This allows for confident quantification and analysis of the spatial localization, distribution and levels of the target proteins using quantitative 3D microscopy.

### Computational modeling of whole cell experimental data

There are two types of experimental data presented in this study. First, we had whole cell data in which spatial information was hidden by summing up the changes in β-catenin within subcellular compartments (see Figure 4A). Secondly we had compartmental data in which β-catenin concentration was monitored in two subcellular compartments (see Figure 4B and C). By removing spatial dependencies, the whole cell data set resembled the single homogeneous system developed by Lee et al. It was then logical, as a first step, to fit a one compartment Wnt model strongly based on the Lee model to this data. This extended the Lee model and is referred to here as Model 1. However, to interpret the compartment data required the development of a multi-compartment model which included inter-compartment transport. Hence, a new simple two compartment β-catenin model, referred to as Model 2 was developed. The results for Model 1 based on whole cell data are presented below.

**Figure 6:**
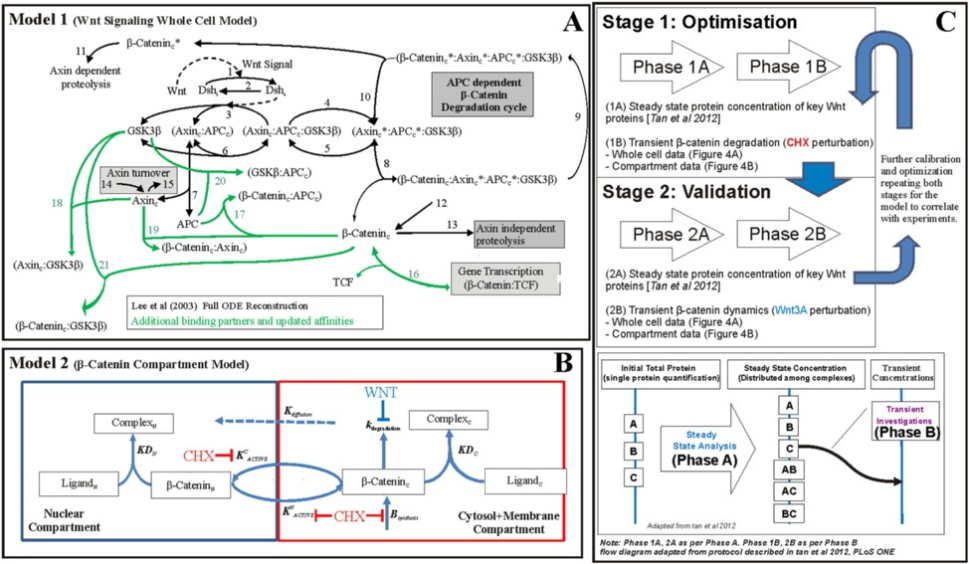
**Schematic diagrams of Computational Models. (A)** Model 1 is a Wnt signaling pathway whole cell model based on the full ODE reconstruction of the Lee et al. (2003) model with alternative binding partners not included and updates on binding affinities for mammalian cells. **(B)** Model 2 is a simple β-catenin compartment model with nuclear and non-nuclear compartments. Model 2 includes a binding partner for β-catenin and considers diffusion and/or active transport mechanisms. **(C)** Schematic of computational modelling stages/phases implemented in this study. Two stages were conducted, each having two phases with the experimental data used during optimization for each phase (1A, 1B, 2A and 2B). (Bottom inset) Phase A, steady state analysis where proteins were distributed among the complexes and Phase B, transient analysis due to specific perturbations.

**Figure 7:**
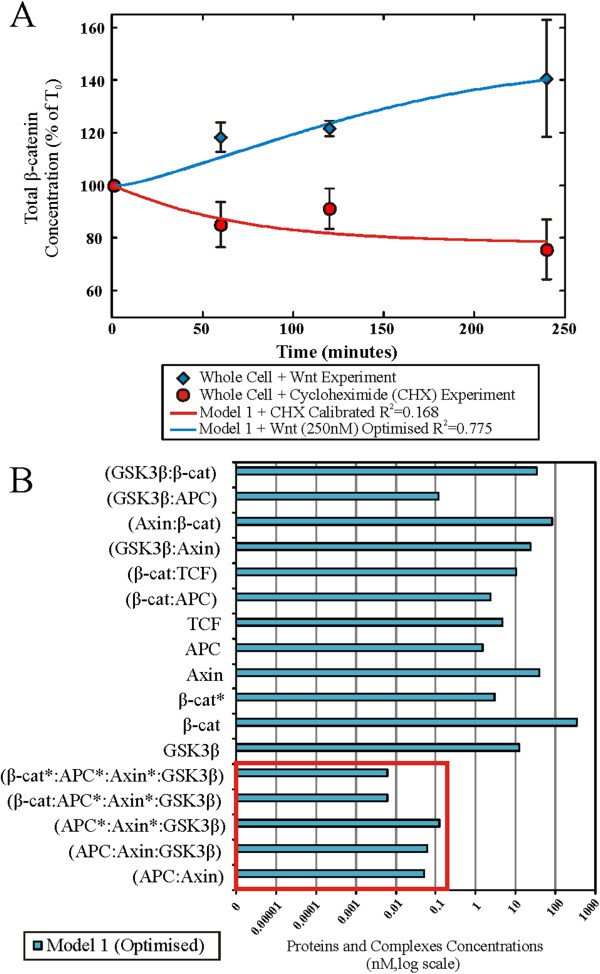
**HEK293T cell model simulations, optimization and results.** Computational optimization of Model 1 for the total β-catenin data obtained using quantitative confocal microscopy (shown in Figure 5). Specifically, **(A)** the model was fitted to the degradation (cycloheximide inhibition experiments in red circles) and Wnt perturbation (blue diamonds) dynamics of total β-catenin. Results show a reasonable correlation between Model 1 and the experiment. **(B)** Redistribution of Wnt signaling pathway proteins and the interacting protein complexes during the steady state using the optimized whole cell Model 1 (log scale). It should be noted that concentrations of protein and complexes in the degradation complex (in red box) are significantly lower than the other proteins (and complexes) in the model.

Our single compartment model (Model 1, Figure 6) was optimized (for selected parameters, see Additional file 1: Table S6) using the steady state total protein concentrations of the key Wnt signaling proteins (i.e. β-catenin, Axin, APC and GSK3β), as measured in our previous study [27], and then fitted to the β-catenin degradation and Wnt3A stimulation dynamics data obtained in this study (Figure 4A). A poor correlation of steady state Axin concentration for Model 1 with the data was observed before optimization (see Additional file 1: Figure S2). During the optimization of the Wnt stimulation data, the Wnt receptor signaling components adjusted included the protein elements Wnt and both (active and inactive) forms of dishevelled. The resulting optimized Wnt model (Figure 7A and see Additional file 1: Figure S2) fitted the experimental data reasonably well with a R^2^ of 0.775 (Figure 4A). The optimization model process and the optimized parameters are described in the Methods section as well as in Additional file 1, Text section 1.6 and Tables S2-S5.

It should be noted, that the optimization of the model came at a ‘cost’, in the form of substantial parametric adjustments (with adjustments of 0.1 to 15000 times the original parameter values from Lee et al., see Additional file 1: Table S6). On further inspection of the protein/complex redistribution profiles during the steady state analysis (Figure 7B), it appeared that the low concentration of the degradation complex (Axin:APC:GSK3β) was the limiting factor in the model when modulating the degradation of β-catenin in the degradation cycle. In Lee et al.’s work, Axin was the component controlling the level of this degradation complex. However in the current study, as the concentration of APC is very low in HEK293T cells [27], the limiting component was APC. In particular, the levels of free APC appeared to be very low at steady state [27].

The perturbation experimental data obtained in this study were a measure of the total β-catenin protein levels for the whole cells (Figure 4A). In Model 1 the only β-catenin-containing component that was clearly present in the nuclear compartment was the β-catenin/TCF complex. The change in the compartment ratio between the nuclear (N, i.e. β-catenin/TCF complex) and non-nuclear compartment (CM, i.e. the rest of the system components) β-catenin intensity was calculated (N:CM, Figure 4C). The predicted N:CM ratio from Model 1 was significantly lower than that of the experiment. Having TCF as the only nuclear compartment binding partner of β-catenin was a likely limitation, as was having the TCF concentration fixed (during model optimization) at the values provided by Lee et al.

Model 1, with parameters optimized to reproduce the whole cell data, was a one compartment, i.e. whole cell model. It was therefore not surprising that optimization to the compartment data was not very successful. However, it did highlight the limitation of the one-compartment model for understanding cell compartment dynamics and a need for developing new models that included multiple cell compartments. In the next section we consider a multi-compartment model (i.e. Model 2).

### Two compartment β-catenin model

Here we show that once diffusion and active transport mechanisms between compartments are included (Refer to Table 1 and Table 2 as well as see Additional file 1 for details of model composition, numerical analysis), the experimental compartment data can be reproduced using a relatively simple two compartment model for β-catenin (Model 2, Figure 6). Various reports [34-41] have suggested transport of β-catenin between the nucleus and cytosol. In this study, a generic active transport mechanism was assumed without specifying the identity of transport protein(s) involved. The model consists of two compartments, one to represent the cell nucleus and another compartment for the cytoplasm-membrane. Note that this two-compartment model corresponded to the spatial resolution of our data. In each compartment it was assumed that β-catenin had a binding partner. This assumption aims to represent the net binding of β-catenin to all possible ligands. The exception to this assumption was the degradation pathway which was treated as a single step via the free (unbound) β-catenin in the cytosol-membrane compartment. We assumed that β-catenin degradation and synthesis only occur in the cytosol-membrane compartment. The free β-catenin was assumed to be transported between compartments by a diffusion-like process (i.e. proportional to concentration difference between compartments) and/or actively (i.e. the rate of transport is independent of the concentration difference between the compartments). The equations describing this model were:

(1) $$\begin{array}{l} \frac{dB^{c}}{\mathrm{dt}}=B_{\mathrm{synthesis}}-k_{\mathrm{degradation}}B^{c}+k_{R}^{C}C^{C}-k_{F}^{C}B^{c}L^{c}\\ \;-\frac{k_{\mathrm{diffusion}}\left(B^{C}-B^{N}\right)+k_{\mathrm{active}}^{C}B^{C}-k_{\mathrm{active}}^{N}B^{N}}{V^{C}} \end{array}$$

(2) $$\begin{array}{l} \frac{dB^{N}}{\mathrm{dt}}=k_{R}^{N}C^{N}-k_{F}^{N}B^{N}L^{N}\\ \;+\frac{k_{\mathrm{diffusion}}\left(B^{C}-B^{N}\right)+k_{\mathrm{active}}^{C}B^{C}-k_{\mathrm{active}}^{N}B^{N}}{V^{N}} \end{array}$$

(3) $$\frac{dL^{c}}{\mathrm{dt}}=k_{R}^{c}C^{c}-k_{F}^{c}B^{c}\left(L_{T}^{c}-C^{c}\right)$$

(4) $$\frac{dC^{c}}{\mathrm{dt}}=-k_{R}^{c}C^{c}+k_{F}^{c}B^{c}\left(L_{T}^{c}-C^{c}\right)$$

(5) $$\frac{dL^{N}}{\mathrm{dt}}=k_{R}^{N}C^{N}-k_{F}^{N}B^{N}\left(L_{T}^{N}-C^{N}\right)$$

(6) $$\frac{dC^{N}}{\mathrm{dt}}=-k_{R}^{N}C^{N}+k_{F}^{N}B^{N}\left(L_{T}^{N}-C^{N}\right)$$

where: the superscripts denote either cytosol-membrane (C) or nuclear (N) compartments. $L_{T}^{N}$ and $L_{T}^{N}$ were the total ligand concentrations in either compartment (C or N). B^C^ and B^N^ were the free β-catenin concentrations in either compartment (C or N), C^C^ and C^N^ were the bound β-catenin-ligand complex concentrations in either compartment (C or N) and L^C^ and L^N^ were the free ligand concentrations in either compartment (C or N). $k_{F}^{N}$ and $k_{F}^{C}$ were the forward rate constants between β-catenin and ligand in either compartment (C or N), with $k_{R}^{N}$ and $k_{R}^{C}$ the corresponding reverse rate constants. B_synthesis_ was the β-catenin synthesis rate, k_degradation_ was the β-catenin degradation rate, k_diffusion_ was the diffusion rate constant and included contributions of the nuclear membrane surface area, effective membrane thickness and diffusion coefficient on the mass flux between compartments by passive diffusion. Finally, active transport between compartments was described using the constants $k_{\mathrm{active}}^{C}$ and $k_{\mathrm{active}}^{N}$ where the superscript related to the compartment from which β-catenin was being transported (‘C’ cytosol-membrane and ‘N’ nucleus).

**Table 1:** Steady state concentrations obtained for optimized Model 2 with and without active transport

**Component**	**Steady state concentration (nM)**		
**-**	**-**	**With active transport**	**Without active transport**
Cytosol-membrane β-catenin	$B_{0}^{C}$	46.6	70.8
Cytosol-membrane Ligand	$L_{0}^{C}$	581.1	605.3
Cytosol-membrane complex	$C_{0}^{C}$	418.9	394.7
Nuclear β-catenin	$B_{0}^{N}$	32.6	61.8
Nuclear ligand	$L_{0}^{C}$	516.8	546.0
Nuclear complex	$C_{0}^{N}$	483.2	454.0

**Table 2:** Change in model parameters for Models 2 after optimization

***Parameters***	***Initial conditions***	***Optimized with active transport***		***Fold change***	***Optimized without active transport***		***Fold change***
**-**	**Steady state**	**CHX**	**WNT**	**-**	**CHX**	**WNT**	**-**
*B*_*synthesis*_	1.306	0	1.306	-	0	1.306	-
*K*_*degradation*_	0. 0163	0.0163	0	-	0.0163	0	-
*kR_*_*cyto*_	0.0026	0.000647		0.25	0.00109		0.42
*kF_*_*cyto*_	0.00001	0.00001		-	0.00001		-
*kR_*_*nuclear*_	0.003	0.00349		1.16	0.00743		2.48
*kF_*_*nuclear*_	0.0001	0.0001		-	0.0001		-
*k*_*diffusion*_	36	39.13		1.09	1354.1		37.6
$k_{\mathrm{active}}^{C}$	4.5	4.5		1	0		-
$k_{\mathrm{active}}^{N}$	2	17.16		8.58	0		-

At steady state the two compartment model required

(7) $$B_{0}^{C}=\frac{B_{\mathrm{synthesis}}}{k_{\mathrm{degradation}}}$$

and that

(8) $$k_{\mathrm{diffusion}}\left(B_{0}^{C}-B_{0}^{N}\right)+k_{\mathrm{active}}^{C}B_{0}^{C}-k_{\mathrm{active}}^{N}B_{0}^{N}=0$$

In the absence of active transport at steady state, i.e. prior to perturbing the system with Wnt3A or CHX, Equation (8) could only be satisfied if $B_{0}^{C}=B_{0}^{N}$, i.e. the free β-catenin at steady state needed to be the same in each compartment. However active transport enabled a difference in free β-catenin to be maintained at steady state. Whether or not free β-catenin was higher in the nucleus or cytosol at steady state, depends on the active transporters and the affinity of the binding partners within each compartment. From our earlier studies [27], initially HEK293T cells had an average total β-catenin concentration of 490nM, using the compartment distribution of β-catenin measured in this study (N:CM of 1.05, Figure 4C at t_0_), the total β-catenin concentration in the cytosol-membrane compartment was calculated as 465.5nM and in the nucleus as 515.8nM. Therefore, in the absence of active transporters, there was a lower bound β-catenin steady state concentration $\left(C_{0}^{C}\right)$ in the cytosol-membrane compartment than in the nucleus $\left(C_{0}^{N}\right)$$\left(i.e.\;C_{0}^{C}<C_{0}^{N}\right)$. Note, to achieve $B_{0}^{C}=B_{0}^{N}$ while $C_{0}^{C}=C_{0}^{N}$ did not restrict the relative binding affinity or total ligand concentration in the cytosol and nucleus. In our model we assumed an excess of β-catenin binding ligands in both the cytosol-membrane and nuclear compartments.

**Figure 8:**
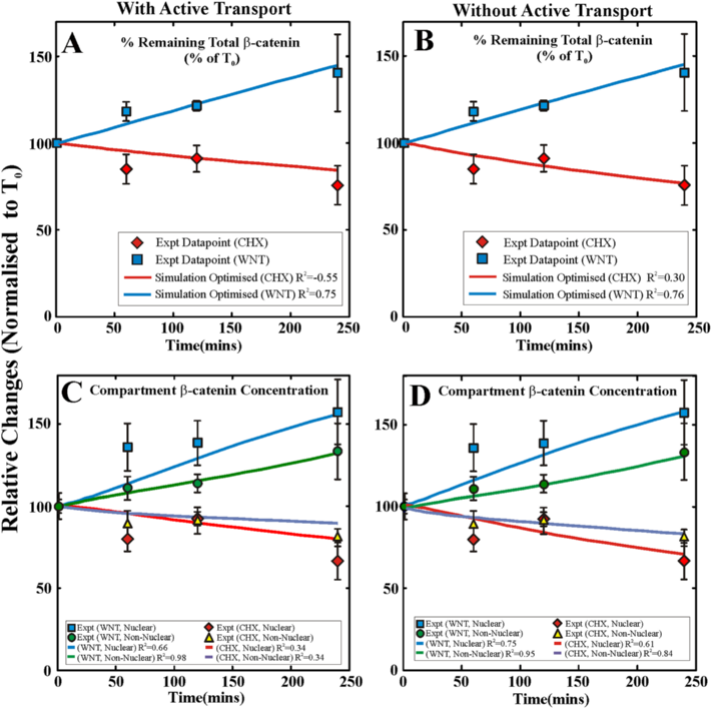
**Role of β-catenin active transport into the nucleus in regulating protein distribution/dynamics in HEK293T cells.** Simulation results from the optimization of the simple two compartment model for β-catenin (Model 2) indicate the critical role of active transport in regulating the compartmental distribution and dynamics of β-catenin in HEK293T cells. Model 2 was optimized for the relative change in total β-catenin concentrations **(A and B)** relative changes in β-catenin compartment concentrations **(C and D)** and relative change in nuclear/cyto-membrane concentration ratios (data not shown). Simulations are conducted with either active transport switch on **(A and C)** or switched off **(B and D)**. Simulations with active transport clearly show an improved correlation and fit to the experimental data when compared with simulations without active transport.

We next consider the transient perturbation data. Our aim was to simulate the compartmental β-catenin changes under CHX inhibition or Wnt3A stimulation using the same model parameters, except with *B*_*synthesis*_ = 0 for CHX inhibition and *k*_*degradation*_ *=* 0 for Wnt3A stimulation. We tested both conditions with (active) or without (passive) active transport and attempted to calibrate the model in each case. Unlike the single compartment model, our optimal solutions (Tables 1 and 2) for both forms of the transport model (passive or active transport), were able to reproduce the Wnt3A and CHX experiments (Figure 8). In the case of active transport, not only was a close reproduction of the experimental compartment results obtained, the efflux diffusion rate for β-catenin from the nucleus was also maintained after optimization (Table 2). This was in contrast to the conditions required to achieve the optimal condition for the passive transport model, where the diffusion rate constant was over 37 times the initial estimated value (see Table 2). Our results suggest that Model 2 with active transport was the better model for Wnt signaling in HEK293T cells.

## Discussion

In previous studies, various mechanisms of Wnt pathway signaling (e.g. the APC-independent β-catenin degradation mechanism) [14,16] as well as cell type specific protein concentration and dynamics have been suggested [27]. There is also an increasing awareness that signaling pathways function across several cellular compartments and so their operation must be understood in this context. Consequently, there is a growing emphasis for recording the spatial localization of proteins and to account for the protein distribution when modeling biological systems [33]. This is evident from the increasing number of multi-compartment and multi-scale computational models of Wnt signaling published in recent years [28-31]. The additional spatial aspect inevitably increases the complexity of the modeling problem; there is an increased number of potential interactions and a requirement for additional data to inform model parameters. At present, this quantitative data is either incomplete or unavailable.

In order to bridge the gaps in the existing data on the cellular distribution of protein components, a protocol using immunofluorescence, confocal microscopy was developed and validated for analysis of the 3D sub-cellular localization of target proteins in whole cells. This protocol allowed the concentrations of target proteins in different compartments of the cells to be determined and changes in concentrations to be analyzed under specific perturbations at different times.

It is difficult to use transfection of fluorescently-tagged proteins (β-catenin in this case), as often the levels, distribution and turnover of the recombinant forms can be quite different to the endogenous proteins. Unless it can be validated that an introduced recombinant protein behaves in a similar manner to the endogenous protein, the use of live cell imaging has limited utility for the study of signaling systems. Therefore, individual formaldehyde fixed samples at specific time points were used. In this case it was essential to have a reference fluorescence probe to normalize the results between the different samples and different time points. We used the InSpeck™ Green beads in each sample as intensity markers.

This imaging quantitation technique provided insights previously unavailable from the cellular extract experiments, in particular protein localization under different perturbations in intact cells. Critically, we observed a distinctly different dynamic behavior for the Wnt signaling proteins in HEK293T mammalian cells than for the Xenopus oocyte cell-free extract. In particular, the rate of β-catenin degradation in HEK293T cells was relative slow (15% decrease in 1 hour compared to a 50% decrease for Xenopus cell free extracts). From the analysis of the transient change in compartment β-catenin concentrations, Wnt stimulation increased the β-catenin concentrations in both the nucleus and cytosol-membrane compartments. Although over 4 hours the rate of increase appeared to be similar in both compartments, an initial rapid increase in β-catenin concentration was observed for the nuclear compartment. This was reflected in the observed rapid increase in the normalized N:CM ratio (∆NCM) in the first hour, before stabilizing at ~1.2 for the duration of the experiment. These results suggest that there is an active transport mechanism, stimulated by Wnt, which concentrates β-catenin in the nuclear compartment. One possibility is that Wnt stimulation increases the activity of potential transporter proteins (e.g. Pygopus, legless/BCL9-2 [34,40], TCF-4 or Menin [35,41]). This increased activity could occur either by a change in the levels or affinity of the transporter proteins or by a change in the affinity of a pool of β-catenin for these transporter proteins (e.g. via specific Wnt associated phosphorylation of β-catenin [18]). Another possibility (which does not require active transport) is that Wnt induces a relative change in affinity for β-catenin for its ligands in the two compartments, thus changing the relative free diffusible β-catenin concentrations (higher in the cytoplasm-membrane compartment than nuclear compartment) and driving β-catenin transport to the nucleus down its concentration gradient. As we were only measuring total (bound and unbound) β-catenin in this study, a change in the ratio of bound and unbound β-catenin was not determined. In any case the similar rate of concentration increment in both nucleus and the cytosol-membrane compartments after 1 hour suggested that drivers for β-catenin transport into the nucleus were balanced by an opposite (passive) diffusion transport out of the nucleus. Note also that under the influence of cycloheximide the rate of loss of β-catenin was the same in both compartments, suggesting that rate of transport between compartments or the rate of release of β-catenin from ligands in each compartment were not the limiting rates for removing β-catenin from the cell. One mechanism to achieve an initial increase in the ∆NCM β-catenin ratio, i.e. a two compartment model, was examined briefly in this study (see below). Our adoption of the two-compartment model does not imply this is the only explanation for the rapid nuclear influx of β-catenin. Different reports have further implicated roles of nucleo-cytoplasmic shuttling of β-catenin and/or its degradation complex binding partners in regulating β-catenin nuclear localization [36-39,41,42]. Firstly, β-catenin has been reported to translocate into the nucleus without having the nuclear localization signal (NLS) sequence, independently of cellular nuclear import mechanisms [36,41]. Secondly, it has been proposed that the APC’s nuclear export signal (NES) allows it to shuttle between the nucleus and cytoplasm, controlling the compartment levels of β-catenin [38,39]. Likewise, it has been reported that a direct interaction of Axin (another β-catenin binding partner) with the CRM1 receptor facilitated its shuttling between the nucleus and cytoplasm [42]. GSK3 has also been reported to have a NLS and binds to FRAT for nuclear export in a CRM1-dependent manner [37]. Interestingly, it has also been reported that GSK3 down-regulates β-catenin activity in the nucleus [43] and that the nucleocytoplasmic distribution of β-catenin is regulated by retention rather than transport [44]. This active transport mechanism and the other possibilities need to be investigated further in subsequent publications. One technique for measuring nucleo-cytoplasmic transport dynamics of proteins is the use of 3D confocal microscopy introduced in this study. Another approach would be to conduct FRAP experiments [44], looking at the recovery of fluorescent-tagged proteins moving back into selectively photo-bleached regions, thus providing data on the motility and rate of movement of these proteins.

Computationally, the total β-catenin kinetic data obtained here were integrated into a previously available Wnt signaling model (Xenopus extract Wnt model from Lee et al. in 2003 [20]) with the aim of developing a Wnt model more representative of mammalian cells (HEK293T). Although in this optimized HEK293T Wnt model (Model 1) dynamics correlated well with the whole cell experimental data, substantial changes to model parameters were required (see Table 1) and this model was unable to capture the compartment data. It was clear that to advance the whole cell model to a spatial compartment model of the Wnt signaling pathway, structural changes and transport mechanisms had to be taken into consideration. To test this hypothesis, a simple two compartment β-catenin model (Model 2) was developed which replicated the experimentally observed β-catenin dynamics. Our results emphasize the importance of creating an appropriate structure for the model, in this case a second cellular compartment is required. While this β-catenin model does not take into account other Wnt signaling components, it provides a good platform for developing a more complete compartment model of the Wnt signaling pathway in HEK293T cells. Moving forward, these predictions will serve as the motivation for specific experiments to be conducted to measure realistic parameter values or bounds/limits for further refinement of the models.

One of the critical decisions when developing the computational models for biological systems was the choice of model parameters. Often experimental measures of these parameters are unavailable, which presents significant obstacles for modeling. Thus the significance of having quantitative measurement techniques such as the one presented in this paper. In cases where no information is directly available, estimations had to be made. In Model 1, the whole cell model, optimization was conducted on parameters that were initially estimated rather than measured experimentally (6 of 21 reactions, Table 1). In Model 2, the two-compartment model, one of these optimized parameters was *k*_*diffusion*_, which corresponds to the transport between the intracellular compartments via diffusion. As no diffusion rate constant (*k*_*diffusion*_) for HEK293T was available, the initial value (*k*_*diffusion*_ *=* 36 m∙min^−1^) was estimated using information from the literature and physics (See Additional file 1, Section 1.8 for details) and based on the transport properties of nuclear pore complexes for proteins [36,45]. When comparing the optimized models in the case of active transport versus diffusion only transport, although both models fit the compartment data, only the active transport model did so without significant changes from our initial model parameter estimates. Specifically it was found that the active transport case had an optimized *k*_*diffusion*_ = 39.13 m∙min^−1^ (i.e. minimal change from the initial estimates) whereas in the case of no active transport *k*_*diffusion*_ = 1354 m∙min^−1^ (i.e. ~37 times larger than initial estimates). This suggests that active transport is likely to be involved in determining the compartment distribution of β-catenin in HEK293T cells under Wnt stimulation. For both conditions of Model 2, the model’s initial response to Wnt3A was too slow. This was evidenced by both sets of simulations missing the nuclear β-catenin concentration data point at 60 minutes for Wnt3A stimulation. The other major change in parameters is that the β-catenin equilibrium constant in the nucleus for both simulations increases (Table 2). The initial value used in this study was inferred from work by Lee et al. [20] for TCF/β-catenin interactions in the Xenopus oocyte. Further validation with experimental data from mammalian sources will be needed to validate this new equilibrium constant to further constraint the model.

These simulation results indicated a closely coupled relationship between the diffusion and active transport rate in the control of β-catenin compartmentalization in this cellular system. We do not discount the possibility that there was likely to be sequestering effects of other partner proteins on β-catenin (e.g. E-cadherin) that will modulate its levels in specific compartments. The simulations presented in this study suggest that a multi-compartment model, in contrast to the homogeneous Lee et al. whole cell model of Wnt signaling (that takes into account compartments and active transport mechanisms) was needed to understand the Wnt3A signaling pathway. Although Model 2 did not take into account the variety of potential binding proteins found in Model 1, using a generic binding partner instead, this simplified β-catenin model provides a good platform for advancing a Wnt signaling model which is more representative of real life systems.

The schematic illustrating Model 2 is similar to that recently proposed by Schmitz et al. [30]. Schmitz and colleagues consolidated the components of the degradation complex (i.e. APC, Axin and GSK3β) into a single species known as AAG and constrained its shuttling rate (v2) with respect to that of the free β-catenin shuttling rate (v1). In this study, Model 2 assumes shuttling of free β-catenin by a diffusion-like or active transport, but no transporter protein is defined explicitly. Another difference between Schmitz et al. [30] and Model 2 is that Model 2 was calibrated with mammalian spatial data. Model 2 was designed to test the need for a multi-compartment approach and whether it might provide further insights on compartment specific Wnt signaling in mammalian cell systems, thus overcoming the limitations of the single compartment models. The Schmitz et al. [30] model was built specifically to study the effect of nucleo-cytoplasmic shuttling on Wnt signaling and as a consequence, they performed a parametric study to explore the possible states of the system. Model 2 primarily aimed to simulate the compartmental dynamic data generated in this study using quantitative 3D confocal microscopy.

One approach for constraining computational models of biological systems is to acquire and optimize for both spatial and temporal data for the signaling components. In most studies, either spatial data or temporal data are available, but seldom are both acquired together. This was one of the key objectives of the methods introduced in this study, to acquire both spatial (compartments) and temporal (time-course) data using immunofluorescence. Computationally, the typical approach has been to use ordinary differential equations and generic rate constants to approximate and optimize these data for simulating complex biological pathways. However, in a biological system, cell growth causes volume changes which could change the associated rate constants in time [46]. As more advanced models starts to incorporate cellular compartments in the time domain, even more approximations would have to be made. In this study, we approximated compartment volumes as the population averages over a steady state population [27] and assumed these volumes do not change in time. Furthermore, we assumed that the rate constants do not change with time. Nonetheless, with the increasing abundance of temporal and spatial data, these considerations would have to be addressed (i.e. time-dependent rate constants and cellular volumes) during the development of the computational models.

Computationally, the optimization process to find the best solution for a complex signaling network is not simple. As the number of parameters in a model increase, the number of permutations to be considered for optimization increases exponentially. As an example, in our study, each model includes four flux terms (two each for Axin and β-catenin) so four unique sequences would require 24 permutations. Approximating each simulation having 1000 random starting points, each search typically requires 100 iterations, a total of 2,400,000 simulation cycles are required. Thus the computational requirements and time will be considerable and constraining. The use of a topological analysis approach (see Additional file 1) helped to reduce the number of iterations needed by making logical deductions when selecting parameters and parametric adjustments. Efforts had been made by many research groups to remove the computational constraints and limitation caused by network size, e.g. by using a rule-based modeling [47,48]. Rule based modeling has the potential to speed up the computational analysis processes involved with pathway simulations and should be explored further as we develop compartmentalized models.

One potential limitation of our experimental system is the number of available confocal channels that can be used for each analysis. Current available microscopes had three to four fluorescent channels, which were shared among monitoring target proteins as well as markers for specific compartments. This study used three channels for both compartment probes and target protein identification. To monitor more target proteins, the procedure had to be repeated for each additional protein of interest. The number of compartments which can be analyzed depends not only on the availability of confocal channels, but also on the suitability/specificity of the marker probes which define the compartments. In this study, two compartments (the nuclei and cytosol-membrane compartment) were identified and analyzed quantitatively. For the study of epithelial cells monolayer or aggregates, it would have been ideal to be able to analyze the membrane compartments separately. Unfortunately, efficient isolation of the membrane fluorescence was limited by membrane marker suitability. Despite many attempts to effectively distinguish and isolate the membrane fluorescence from the cytosol, we were unable to resolve the boundaries between neighboring adherent cells at various depths **in 3D space**. Consequently, our study is limited to two compartments, but with further investigation into image processing techniques and improved membrane markers, it should be possible in subsequent studies to analyze three compartments (nucleus, cytosol and membrane) for each cell in a monolayer or even an aggregate of cells.

We note that the compartment concentration distribution of β-catenin reported here does not correlate with our previously reported β-catenin distributions using sub-cellular fractionation technique [27]. The sub-cellular fractionation procedures applied in the previous study recovered the majority of the compartment proteins (well fractionated), but there were inevitable losses associated in each step of the process. Loss was associated with pipetting, washing and mechanical disruptions of the cell and transfer of fluids. In particular cytoplasmic proteins were lost during the isolation of the other compartments. In contrast, the microscopy technique was able to give a more accurate quantitation of the protein concentrations as it measures the proteins concentrations “in situ” without external intervention or cellular disruptions. In comparison to sub-cellular fractionation, the confocal imaging quantitation technique therefore provides a more realistic and accurate measurements of the protein concentrations in each compartment.

Other components of the cell also modulate the distribution and function of β-catenin. For example, the Cadherin family proteins are key adhesion partners of β-catenin in the cell-cell adhesion processes [49,50]. These interactions will influence the distribution of β-catenin within the cell. From the immunofluorescence images, it was clear that a substantial amount of β-catenin was localized at the membrane (presumably with a cadherin). This observation, coupled with recent reports of differing modes of action of Wnt signaling on β-catenin (e.g. modulates ubiquitination of β-catenin [19]) as well as distinct pools of Wnt induced β-catenin isoforms at different locations in the cell [17,18], provides further incentive to develop a multi-compartmental experimental and modeling configuration to describe the signaling processes within a cellular network.

## Conclusions

In this study, we have developed and applied an integrated experimental computational approach to investigate the spatial and temporal dynamics of Wnt signaling in HEK293T cells. A 3D quantitation protocol using 3D confocal microscopy, immunofluorescence and image processing was developed to acquire quantitative cellular compartment (nucleus and cytosol-membrane) and temporal data for target protein β-catenin concentrations under different signaling perturbations. Our results suggest that β-catenin diffusion between compartments is faster than β-catenin degradation in the cytosol and that Wnt stimulation increases total β-catenin throughout the cell. Initially (~first hour), there is a faster increment of β-catenin in the nucleus. This experimental data was used to parameterize mathematical models of Wnt signaling in HEK293T cells. Computational analysis comparing a single and a two compartments model were conducted. Both models were able to reproduce whole cell changes in β-catenin. However, only the two compartment model could reproduce the Wnt3A induced β-catenin changes, and the best reasonable fit was obtained with the inclusion of an active transport component alongside passive diffusion to account for the rate of transfer of β-catenin to the nucleus. This suggested a role of active transport and compartmentalization in regulating both the spatial and temporal dynamics of β-catenin when HEK293T cells are stimulated by Wnt3A.

## Methods

### Antibodies, reagents and intensity standards

The antibodies used in this study were: mouse monoclonal anti-β-catenin (BD Transduction Laboratories, BD Biosciences, San Jose, CA, cat#610153) and mouse monoclonal anti-N-cadherin (BD Transduction Laboratories, BD Biosciences, San Jose, CA, cat#610921). The anti-β-catenin marks the target protein endogenous β-catenin in the cell while the N-cadherin was used both as a marker to outline the whole cell as well as to stain the endogenous N-cadherin protein in the cell. The fluorescent stain 4, 6-diamidino-2-phenyl indole Nucleic Acid Stain (DAPI, cat# D1306 Molecular Probes Inc, Eugene, OR, Invitrogen) was used to stain the nuclei. The secondary fluorescent dyes used were Alexa Fluor ®488 goat anti-rabbit IgG (H + L) (Molecular Probes Inc, Eugene, OR, Invitrogen cat#A-11034) and Alexa Fluor® 546 goat anti-rabbit IgG (H + L) (Probes Inc, Eugene, OR, Invitrogen cat#A-11030).

Perturbation of the Wnt pathway was conducted using a known activator/stimulator of the Wnt signaling pathway [51], Wnt3A (Wnt3A CM, in the form of L-cell conditioned media containing the Wnt3A ligand was acquired from L-cells producing and secreting the recombinant Wnt3A protein, LWnt3A cell (ATCC#CRL-2647, [52,53]). A known inhibitor of protein biosynthesis, cycloheximide (CHX, Sigma-Aldrich Corporation, St. Louis, MO 63103 USA cat#C7698 [54]) was purchased from Sigma-Aldrich. 4% formaldehyde (Formaldehyde solution min. 37% free from acid, cat#103999, 64293 Darmstadt, Germany) in phosphate buffered saline (PBS) was used as a fixation agent to preserve the sub-structures of the cells (physically and chemically) for microscopy.

InSpeck™ Microscope Image Intensity Calibration Kits, specifically 6.0 μm InSpeck™ Green (cat#I-14785, Molecular Probes Inc, Eugene, Oregon), were utilized as the intensity reference for confocal microscopy. 0.3% rated microspheres were identified for use in these experiments based on a selection criteria outlined in Additional file 1 main text, Figure S3 and Table S1.

### Cell culture, sample preparation and treatments

HEK293T, a human kidney epithelial cell line [33] was used in this study as these cells are responsive to Wnt3A stimulation and have no known mutations among the proteins associated with Wnt signaling. The cells were grown in Dulbecco’s modified Eagle’s medium (DMEM) supplemented with 10% fetal calf serum (FCS) on glass cover slips. For Wnt pathway investigations, the respective cover slips of cells were incubated with either Wnt3A conditioned media (40% in DMEM + 10% FCS) or cycloheximide (CHX, 100 μg/ml in DMEM + 10% FCS) for 0, 1, 2 or 4 hours selectively at 37˚C. Wnt3A is an activator of the Wnt pathway which has been reported to increase the levels of target protein β-catenin in the cell [51], while CHX is a protein synthesis inhibitor [54] which allows for monitoring of the degradation of existing pool of β-catenin protein in the cell in the absence of new synthesis. At the end of the respective incubation periods, the cover slips of cells were fixed with 4% formaldehyde (Formaldehyde solution min. 37% free from acid, cat#103999, 64293 Darmstadt, Germany) in PBS. The fixed cell samples were then immuno-stained with primary anti-β-catenin and anti-N-cadherin antibodies, secondary antibodies Alexa Fluor® 488 and Alexa Fluor® 594 and fluorescent dye DAPI for immunofluorescent studies under the confocal microscope. The stained samples were stored in HT-PBS at 4°C until just before confocal imaging.

### 3D confocal imaging and quantitative compartmental analysis

The fixed and stained cell samples were mounted onto Sykes Moore Chambers (Bellco Glass Inc., Vineland, NJ) with an aliquot of InSpeck™ intensity microspheres PBS mixture and allowed to settle. As this was a fixed time-course system with independent samples of cells on a different cover slip, the InSpeck™ intensity microspheres were used as the intensity reference to facilitate comparisons between the samples (see Additional file 1: Text and Figure S4 for details).

An Olympus FV1000 confocal microscope with a 60x water immersion lens was used to acquire 3D image stacks of the stained cells which were then processed using Metamorph Premier image processing software into image data files. DAPI, β-catenin and N-cadherin fluorophores on the antibodies were excited with the 405 nm, 488 and 594 nm laser lines respectively and the emission captured at wavelengths 405 nm, 473 nm and 559 nm, respectively. Image analysis procedures for InSpeck™ intensity calibration and compartmental analysis were performed on the resulting data files (in Matlab version R2007a (The Mathworks Inc.)) to obtain the resultant β-catenin measurements for each sample. In this study, either Wnt3A or the inhibitor CHX was added to the cells and spatial 3D image stacks acquired 0, 1, 2 or 4 hours after adding the reagent.

Note that for compartmental analysis, only two sub-cellular compartments (nuclei and cytosol-membrane) are defined. Experimentally it was not possible to distinguish the membrane compartment from the cytosol, primarily due to interferences from neighboring cells in 3D space. With improved image processing and membrane markers, subsequent studies will be aimed at progressing to a three compartment model.

### Validation of total whole cell β-catenin confocal measurements using quantification western blots

Quantitative western blots were conducted to validate the 3D microscope fluorescence intensity quantitation technique. Specifically, HEK293T cell cultures were plated in 6 well plates with DMEM + 10% FCS and cultured overnight (until about 70 ~ 80% confluent). The media was removed and replaced with new media containing Wnt3A (40% Wnt3a L-cell CM in DMEM + 10% FCS) or CHX (100 μg/ml) for respective wells at predefined times. The cells were incubated for the required time intervals (0, 1, 2 or 4 hours at 37˚C). HEK293T cultures had been incubated with either Wnt3A or CHX for the 0, 1, 2 or 4 hours at 37°C (i.e. similar to confocal quantification experiments).

At the end of incubation, the cells were harvested, lysed in lysis buffer on ice containing 1% triton-X100 and 1% deoxycholate, and the debris spun down at 13000 rpm for 10 minutes at 4°C. The cell extract was collected as the whole cell lysate and stored at −20˚C. Quantitative SDS-PAGE was conducted with SDS sample buffer (4x, NuPAGE LDS Sample Buffer Cat#NP0007, Invitrogen) and DTT using NuPAGE 4-12% Bis-Tris Gels. In the analysis, recombinant β-catenin proteins were loaded in the same gel to form the standard curve for quantitation.

Proteins were transferred to Nitrocellulose or PVDF membranes and immuno-blotted with primary mouse monoclonal anti-β-catenin, mouse monoclonal anti-β-tubulin antibodies (BD PharMingen™, BD Biosciences, USA, cat#560381) and secondary antibody (Goat anti-mouse IRDye® 800CW, Li-COR Biosciences, and USA cat #926-32210). The resulting blot was scanned using the Odyssey infra-red scanner at 800 μm and results analyzed using the LI-COR Odyssey™ application software (version 3.0, LI-COR, Inc., Lincoln, NE, USA), quantifying the protein band intensities and normalizing with the respective β-tubulin intensities (as control). The quantitative western blots results were compared to the confocal 3D-measurements.

### Computational models

The computational models developed in this study are referred to as Models 1 and 2 (Figure 6A and B). Model 1 was a single compartment model (Figure 6A), developed using a full ODE reconstruction model of Lee et al. 2003 Xenopus Wnt signaling model [21] and extended by including additional protein complexes not found in the Lee et al. model. Model 2 (Figure 6B) was a simplified two compartment β-catenin model, with one compartment representing the cell nucleus and the second compartment representing everything else and was referred to as the cytosol-membrane compartment.

#### Model 1: single compartment Wnt pathway model

Model 1 extends the Lee et al. model to also account for additional potential permutations of the key proteins complexes (specifically APC:β-catenin, Axin:β-catenin, GSK3β:β-catenin and GSK3β:Axin). The following reactions had been added, V18: binding of GSK3β with APC (see Additional file 1: Text and Table S2), V19: binding of β-Catenin with Axin [7,55], V20: binding of β-Catenin with GSK3β (see Additional file 1: Text and Table S2), V21: binding of GSK3β with Axin [6,55]. Furthermore, two reactions (V17: binding of β-Catenin with APC, V18: binding of β-catenin with TCF) have been updated using recently obtained unpublished mammalian binding affinity data (see Additional file 1: Text and Table S2). For the SBML file, see Additional file 2.

#### Model 2: two compartment β-catenin model

To describe the two compartment imaging data detailing changes in β-catenin concentration under Wnt and CHX, we developed a simplified model of this system. The aim was not to develop a complex Wnt signaling model containing all the potential protein-protein interactions and transport, synthesis and degradation processes. A common criticism of this approach has been that with so many unknown adjustable parameters it can easily fit any experimental data. Instead, we use a simplified model which groups multiple processes into generic interactions and use this system to guide us towards understanding what general processes need to occur within the cell and within the Wnt pathway for models to reproduce the experimental data. However simply stated, the model consists of a nuclear and a cytosol-membrane compartment. Each compartment has β-catenin interacting reversibly with a single binding partner. β-catenin is synthesized and degraded only in the cytosol-membrane compartment, however it can be transported between compartments by passive diffusion and active transport. The schematic of the equations are as shown in Figure 6 and see Additional file 3 for the SBML file. (Details of model equations, numerical solutions and simulations are as described in the Tables 1 and 2, Additional file 1: Text and Table S7).

#### Model parameter calibration

The computational models were constructed in the full ‘ODE’ (ordinary differential equation) form and solved in Matlab version R2007a (The Mathworks Inc.) using the stiff solver ‘ode23s’ [56]. Model optimization was conducted using Matlab’s cost-minimization function ‘*patternsearch’* with a cost function based on the coefficient of determination, R^2^. Experimental and computational integration involved two stages (Figure 6C). Stage one was the optimization of model parameters to a set of experimental data calibrations (both steady state and transient data). After the model calibration, stage two involved the validation of the calibrated model using a set of test transient experimental data.

Each stage required two modeling phases (see Figure 6C) as described previously in ref [27]. Phase A calibrated the model for the conservation of total protein concentration during protein redistribution into various protein complexes. Using experimentally measured steady state total protein concentrations as inputs, this phase obtains a stable initial steady state for modeling. In this study, steady state protein concentrations of key Wnt proteins from ref [27] were used for Phase A. Phase B optimized the model to reproduce the transient behavior of the system observed experimentally.

In Stage 1A of this study, the model calibration used the total protein concentration as the initial and objective concentrations, while the Stage 1B model optimization used the transient β-catenin degradation from CHX perturbation (either whole cell data (Figure 4A) or 2 compartment data (Figure 4B)). Stage two (model validation) required both Phase 1A and 1B to be optimized for the calibration data (set as a prerequisite). In Stage 2A and 2B, using the optimized model, validation was conducted with the transient Wnt3A perturbation data (either whole cell data (Figure 4A) or two compartment data (Figure 4B)). Based on the results of the validation, further calibration and optimization of the model was carried out (repeating both stages) in order for the model to be consistent with the experimental data (total protein concentrations and both transient perturbation datasets).

In Phase B, for β-catenin degradation, protein synthesis rates were set to zero, while for Wnt perturbation, Wnt concentrations and inactive dishevelled were set to the initial concentrations of 1nM and 100nM respectively (as per Lee et al. (2003) [20]). In order to identify the best possible parameters for adjustments, sensitivity, steady state and topological, analyses were applied in support of the optimization (see Additional file 1).

## Competing interests

The authors have declared that no competing interests exist.

## Authors’ contributions

CT, YH and AWB conceived and designed the experiments. CT and YH performed the experiments. CT, YH and AWB analyzed and interpreted the experimental data. CT and BSG conducted the computational modeling. CT, BSG and DWS evaluated the computational model results. CT drafted the manuscript. CT, BSG, DWS and AWB participated in revising the paper. All authors have read and approved the manuscript.

## Supplementary Material

Additional file 1 **Supplementary Materials.** This file provides further details of confocal imaging protocols and computational processing analysis utilized in this study. Documentation includes Supplementary Text sections 1.1-1.10, **Tables S1-S7** and **Figures S1-S5**. Click here for file

Additional file 2 **SBML file for Model 1.** The SBML file Model1.xml can be browsed using JDesigner of the Systems Biology Workbench. Please download JDesigner at http://sbw.sourceforge.net/, install it, and open the SBML file Model1.xml to browse the Wnt signaling model. Click here for file

Additional file 3 **SBML file for Model 2.** The SBML file Model2.xml can be browsed using JDesigner of the Systems Biology Workbench. Please download JDesigner at http://sbw.sourceforge.net/, install it, and open the SBML file Model2.xml to browse the Wnt signaling model. Use the ‘Manage Parameter Sets’ option to switch between parameters for Model 2 (active) and Model 2 (passive). Click here for file
