# Supplementary material for: Analysis of Wnt signaling β-catenin spatial dynamics in HEK293T cells
Source: BMC Syst Biol. 2014 Apr 8;8:44. doi: 10.1186/1752-0509-8-44 (PMC4108056; doi:10.1186/1752-0509-8-44)
Supplement: Additional file 1 — Supplementary Materials. This file provides further details of confocal imaging protocols and computational processing analysis utilized in this study. Documentation includes Supplementary Text sections 1.1-1.10, Tables S1-S7 and Figures S1-S5. [file 1752-0509-8-44-S1.pdf]

# Supplementary Materials

# Analysis of Wnt signaling β-catenin spatial dynamics in HEK293T cells

**Chin Wee Tan[1, 2, 3, 4, §], Bruce S. Gardiner[5], Yumiko Hirokawa[1, 3], David W. Smith[5], Antony W. Burgess[1,2,3,6]**

1 Structural Biology Division, The Walter and Eliza Hall Institute of Medical Research, Parkville, Victoria, Australia

2 Department of Medical Biology, University of Melbourne, Parkville, Victoria, Australia

3 Ludwig Institute for Cancer Research, Melbourne-Parkville Branch, Parkville, Victoria, Australia

4 Department of Infrastructure Engineering, University of Melbourne, Parkville, Victoria, Australia

5 School of Computer Science and Software Engineering, The University of Western Australia, Perth, Western Australia, Australia

6 Department of Surgery, University of Melbourne, Parkville, Victoria, Australia

§Corresponding author

# Table of Contents:

## *Confocal Imaging Protocols and Computational Processing Analysis*

1.1 Intensity Microsphere Selection ....................................................................4
1.2 Confocal 3D Imaging Acquisition and Procedures ....................................................5
1.3 Intensity Microspheres Calibration....................................................................6
1.4 Image Processing and Compartmental Analysis..........................................................7
1.5 Computational Modeling Optimization Tools..............................................................9
1.6 Model 1 Optimized Equations and Parameters..........................................................10
1.7 Model 2 (Simplified Two Compartment β-catenin Model) Numerical Analysis...............12
1.8 Estimation of initial conditions and parameter values.........................................13
1.9 Model Equations and Analysis ..........................................................................14
1.10 Model 2 Numerical optimization and results..........................................................17

## Supplementary Tables

Table S1. Flow Cytometry Analysis of InSpeck™ microspheres. ...........................................4
Table S2. Optimized Parameters for Model 1. ..........................................................10
Table S3. Initial and Steady State Concentrations (Optimized) for Model 1. ........................11
Table S4. Reaction rate equations and associated variables for Model 1..............................11
Table S5. Rate of change equations for each species (Model 1). ...........................................12
Table S6. Selected parameters of the model 1 adjusted in this study.....................................12
Table S7. Initial starting parameters for Models 2. ....................................................14

## Supplementary Figures

Figure S1. HEK293T cytosolic extract β-catenin degradation experiments. ............................3
Figure S2. HEK293T Cell Model, integration and optimisation of Model 1. ...........................3
Figure S3. Flow Cytometry Analysis of InSpeck™ Green intensity microspheres. .................5
Figure S4. Microsphere measurements for HEK293T cells labeled with β-catenin.................6
Figure S5. 3D Compartmental Analysis of 3 fluorescence signals in HEK293T cells. ............9

## Additional Figures

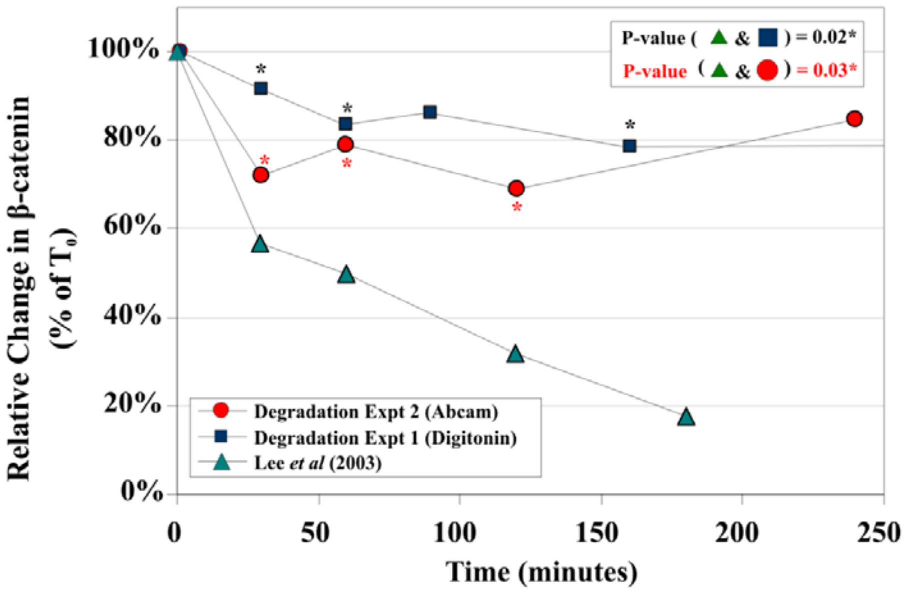


**Figure S1. HEK293T cytosolic extracts: β-catenin degradation experiments.**
Comparison of β-catenin degradation in two experiments analysing cytosolic fractions from HEK293T. The results are compared to the reported β-catenin degradation in Xenopus oocyte cytosolic extracts (Lee *et al* (2003)). Blue squares: Digitonin/EDTA fractionation; Red circles: EDTA/without Digitonin (Abcam); Green triangles: Xenopus oocyte extracts. Anova tests were conducted for Digitonin against Lee *et al* for t=30, 60 and 120 mins; Abcam against Lee *et al* for t=30, 60 and 160/180 minutes (see top insert).

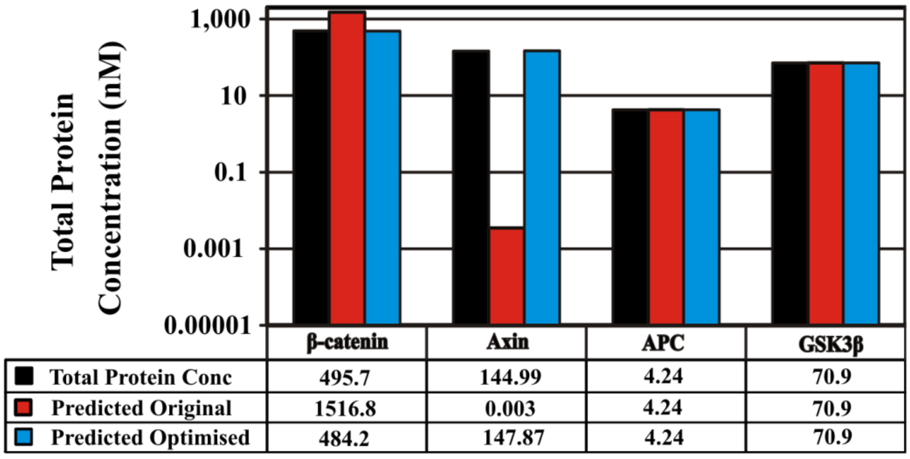


| | β-catenin | Axin | APC | GSK3β |
|---|---|---|---|---|
| Total Protein Conc | 495.7 | 144.99 | 4.24 | 70.9 |
| Predicted Original | 1516.8 | 0.003 | 4.24 | 70.9 |
| Predicted Optimised | 484.2 | 147.87 | 4.24 | 70.9 |

**Figure S2. HEK293T Cell Model, integration and optimisation of Model 1.**
Models 1 steady state (SS) protein concentrations showing the initial protein concentrations (obtained from ref.[1]) and predicted SS total concentrations before and after optimization. Results for key proteins β-catenin, Axin, APC and GSK3β are shown. The predicted totals for both β-catenin and Axin are significantly different from the objective total before optimization. After optimization, the total protein concentrations of the proteins were maintained. Note that APC and GSK3β totals are conserved in the model so no difference is expected.

# Confocal Imaging Protocols and Computational Processing Analysis

### 1.1 Selection of Microsphere Intensity

The system used in this study is a fixed time-course with each time-point represented by an independent sample of cells on a different cover slip. This is unlike time-dependent live cell monitoring systems, where the different time points are from the same sample. Consequently, there may be variability in confocal image intensity due to fluctuations in equipment. To compare quantitatively between the samples at different time points, a common intensity reference point was established for the different samples. Intensity microspheres were employed as a reference to determine and correct for variations between the samples under experimental conditions. The intensity references used in this study were the InSpeck™ Microscope Image Intensity Calibration Kits, specifically 6.0µm InSpeck™ Green (cat#I-14785, Molecular Probes Inc, Eugene, Oregon), which were 6.0 µm diameter polystyrene fluorescent microspheres with a range of relative intensities.

To determine a suitable working range for microscopy of the InSpeck™ microspheres, five different relative intensity rated microspheres (0.3%, 1%, 3%, 10% and 30% rated) were analyzed using a FACS Calibur (Becton & Dickinson, Mountain View, CA, USA) to determine their *actual* fluorescence rating (verified with values supplied by the manufacturer). Results (Figure S3A) show that microspheres intensity was specific with distinct sharp peaks obtained for each rating with a coefficient of variation (CV %) for each category rating found to be usually less than 10% (Table S1). Importantly, the relationship between intensities of different ratings (provided by the manufacturer) and the (FACS) measured intensities of the InSpeck™ intensity microsphere was linear (coefficient of determination of $R^2$=0.9991, (Figure S3B). Therefore, labeled mean intensities were applied as a reference to mean intensities of the microspheres for quantification.

**Table S1. Flow Cytometry Analysis of InSpeck™ microspheres.**
Results of FACS Calibur measurement for different categories of microspheres. CV% was calculated from each individual category rating by dividing standard deviation with the measured mean intensity. As indicated on each vial the labeled rating is measured by the manufacturer.

| ***InSpeck™ Category Rating*** | ***InSpeck™ Mean Microsphere rating provided by manufacturer*** | ***Measured Mean Fluorescent Intensity (FACS)*** | | |
|---|---|---|---|---|
| **(%)** | **(%, Labeled)** | **Mean Intensity** | **Standard Deviation** | **CV%** |
| 0.3 | 0.41 | 42.5 | 3.1 | 7.2 |
| 1 | 1.2 | 121 | 10.4 | 8.6 |
| 3 | 3 | 324 | 25.6 | 7.9 |
| 10 | 8.1 | 903 | 70.4 | 7.8 |
| 30 | 44 | 4219 | 312.0 | 7.4 |

Similarly, confocal intensity quantitation of the four known ratings of microspheres (InSpeck™ 0.3%, 1%, 3%, 10% category rated) was conducted to generate a standard curve for the intensity against ratings and compared with the estimated relative intensity using the FACS Calibur to ascertain a suitable rated microsphere for this experiment. Results for total intensity of each 3D microsphere quantified and tabulated (Figure S3C). Figure S3C showed the standard curve between measured (confocal) mean InSpeck™ Integrated Intensity ($II_{mean}$) against its intensity ratings, showing that the InSpeck™ microspheres had a linear fluorescence intensity (linear $R^2$ fit = 0.9986) in agreement with the flow cytometry results (Figure S3B). The 0.3% rated InSpeck™ microspheres were selected as intensity calibration standards for standardizing/normalizing the intensity levels between samples and time points.

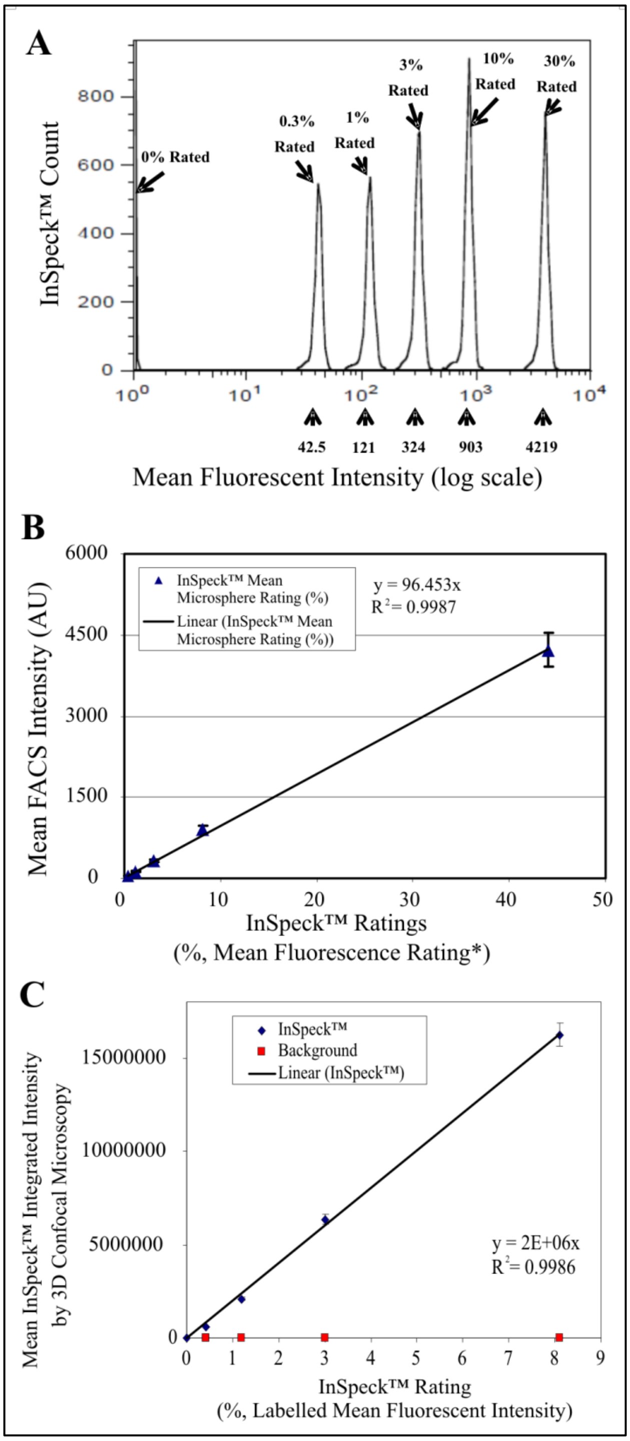


**Figure S3. Flow Cytometry Analysis of InSpeck™ Green intensity microspheres.**

(A) Distinct peaks observed for each vial of microspheres. Each peak represents a different intensity group of microspheres with measured sample mean intensity fluorescent of 42.5 (0.3% rated), 121 (1.0% rated), 324 (3.0% rated), 903 (10% rated) and 4219 (30% rated). Standard deviations and CV% of each sample are given in Table S1. (B) Standard curve of FACS measured mean fluorescent intensity against mean fluorescence rating provided by the manufacturer of the InSpeck™ microspheres. A linear relationship was obtained between the rating and the measured fluorescence intensities with a coefficient of determination $R^2$ of 0.9991. (C) Standard curve for 3D confocal microscopy quantification of InSpeck™ microspheres with the measured mean integrated intensity ($II_{mean}$) of different intensity rated microspheres against actual mean fluorescent intensity (labeled) ratings. Integrated intensities of the background image signal indicate minimal background noise.

## 1.2 Confocal 3D Imaging Acquisition and Procedures

A suspension of the 0.3% InSpeck™ microspheres in HT-PBS was prepared (5µl of stock per mL PBS, ~20000 microspheres/mL), and kept in the dark. Glass slides of stained cell cultures were mounted onto Sykes Moore Chambers (Bellco Glass Inc., Vineland, NJ). The InSpeck™ PBS mixture was homogenized and an aliquot (~200µl) was added to the samples in the chambers covering the samples. The samples were kept out of the light and allowed to settle for 5 minutes.

The confocal imaging procedures were based on the earlier description [1] and in the main text. The images were captured using Olympus FluoroView software (Version 1.7c). Regions of interest (ROI) were selected based on the criteria that two or more stationary InSpeck™ microspheres were in the field of view, the microspheres were not in contact with the samples (isolated) and the samples were monolayer and with minimal surrounding fluorescent debris. 3D image stacks were acquired, encompassing most of the depth of the sample in the ROI. The 3D z-stacks were made up of 2D sectional images with a cubical voxel/pixel size of 0.3 µm$^3$ at 84x magnification to ensure optimal axial sampling. Each 2D sectional image in the z-stack was captured as a fluorescence image of 512 x 512 pixels digitized to 16 bits resolution (65536 levels of grey) representing the intensities detected and saved as an Olympus Image Binary (OIB) image. Triplicate sets of z-stack images were acquired for each time point. Furthermore, image stacks consisting of only InSpeck™ microspheres

(microsphere-only stacks) were acquired to serve as reference microsphere intensity measurements. Image analysis procedures for InSpeck™ intensity calibration and compartmental analysis were performed in Matlab [2] to obtain the resultant β-catenin calculations for each individual sample.

### 1.3 Intensity Microspheres Calibration

Two groups of microspheres were imaged during the experiments. The first group was calibration microspheres (typically two or more) scattered among the primary cell samples (see Figure S4A). These were used as intensity references for each image stack. The second group was reference microspheres used as background references. Reference microspheres were acquired from microsphere-only stacks, that is, images which contained microspheres in media with little or no cells (see Figure S4B). Individual microsphere intensity data was acquired as a 3D image stack using Metamorph Premier (v7.6.3.0, MDS Analytical Tech.,[3]) by drawing circles around each microsphere using the graphical interface function ‘region’ of Metamorph. The “region measurements” function in Metamorph was subsequently applied on the individual image stack of the intensity microsphere to acquire the total intensity of each microsphere by summing up each section of the image stack (see Figure S4C). The total intensity for each microsphere was tabulated and exported into Matlab and the average intensity per microsphere calculated. Integrated Intensity (*II*) levels were calculated from the average total intensity per microsphere for each 3D image stack. As the microspheres were scattered randomly, this ensured an unbiased selection of image Regions of Interest (ROI). ROI selections were based on areas of the sample with two or more isolated (randomly scattered/positioned) microspheres in the field of view.

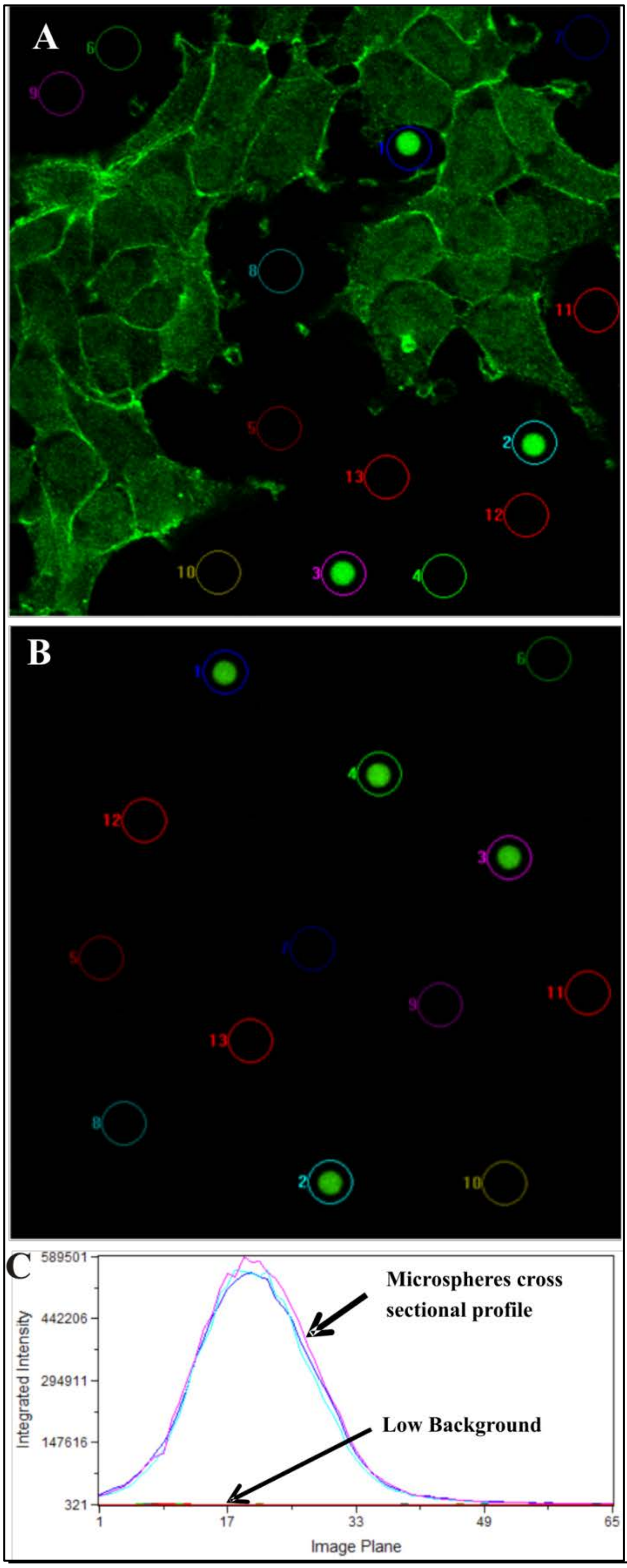


**Figure S4. Microsphere measurements for HEK293T cells labeled with β-catenin.**

Image sections of β-catenin with/without microspheres (green spheres) were processed in Metamorph. Regions are drawn around each microsphere in the image stack for stacks (A) with stained HEK293T cells and (B) without stained cells (background only). (C) Cross-sectional (z-axis) intensity profiles of each microsphere are visually inspected to ensure the microspheres are independent and the background is clear of debris. 3D intensities for each microsphere on each image plane was calculated and exported.

The ‘Reference Integrated Intensity level ($II_{ref}$)’ of the reference microspheres was the Integrated Intensity (*II*) calculated from microsphere-only image stacks for each particular imaging session. Variation in $II_{ref}$ was kept below the averaged variation of the microspheres ($II_{STD}$) determined in Section 1.1 by filtering out image stacks with more than 20% variation in the microsphere intensity. Similarly, the Mean InSpeck™ Integrated Intensity ($II_m$) was calculated from cell sample stacks. The normalized relative microsphere intensity ($II_{norm}$) for each cell stack was calculated from $II_m$ with reference to the $II_{ref}$ (% of $II_{ref}$).

Image stack selection/filtering was subsequently conducted based on this $II_{norm}$ within a variation range of between ± 20% to 30% to maintain the variation from $II_{ref}$ to within $II_{STD}$. Image stacks with highly variable microsphere levels (<80% or >130% of $II_{norm}$) were discarded. The resulting $II_{norm}$ was used in the compartment analysis to normalize the samples. This microsphere calibration procedure limited the variability between the independent samples as well as correcting for any variations that occurred within the intensity range of the microspheres.

### 1.4 Image Processing and Compartmental Analysis

The image stacks were imported into Matlab and the signals from the different fluorescence channels separated. Specifically, DAPI (blue), β-catenin (green) and N-cadherin (red) channels were separated into their respective mono-color stacks (Figure S5 Top Panel). Using the DAPI and N-cadherin signals stacks, selective filtering and image intensity thresholds were applied to isolate the region of interest (Figure S5 Middle Panel, DAPI marks the nuclei compartment while N-cadherin outlines the whole cell). This generated the 3D binary compartment mask to be applied over the target (β-catenin) 3D signal channel to evaluate the co-localization of the β-catenin intensities in the respective compartments. Two compartments (the nuclear and cytosol-membrane compartment) were defined and used in this study. The total 3D intensities of β-catenin co-localizing in each compartment were calculated, along with the volume of each compartment (based on the total number of pixels in the compartment). This data were consolidated for all image stacks and processed (Figure S5 Bottom Panel). Calculations of the relative changes in β-catenin levels due to the effects of either Wnt3A or CHX were calculated for the nuclei (Equation S1), cytosol-membrane (Equation S2) and whole cell compartments (Equation S3). The changes in the compartment β-catenin intensity concentrations and the relative ratios of the β-catenin levels between the two compartments (Equation S4, $Ratio_{\beta\text{-cat,(Nuclei vs. Cyto-Memb)}}$) were also calculated.

$$Conc_{\beta-\text{cat,Nuclear}} = \frac{\Sigma I_{\text{Nuclear}}}{\Sigma V_{\text{Nuclear}}} \times II_{\text{norm}}$$

**Equation S1**

$$Conc_{\beta-\text{cat,Cyto}-\text{Memb}} = \frac{\Sigma I_{\beta-\text{cat,Cyto}-\text{Memb}}}{\Sigma V_{\beta-\text{cat,Cyto}-\text{Memb}}} \times II_{\text{norm}}$$

**Equation S2**

$$Conc_{\beta-\text{cat,WholeCell}} = \frac{\Sigma I_{\beta-\text{cat,WholeCell}}}{\Sigma V_{\beta-\text{cat,WholeCell}}} \times II_{\text{norm}}$$

**Equation S3**

$$Ratio_{\beta-\text{cat,(Nuclear vs. Cyto-Memb)}} = \frac{Conc_{\beta-\text{cat,Nuclear}}}{Conc_{\beta-\text{cat,Cyto-Memb}}}$$

**Equation S**4

The relative concentration of β-catenin in the nuclear compartment ($Conc_{\beta\text{-}cat,Nuclear}$) was calculated from $\sum I_{Nuclear}$ (the summation/total intensity of β-catenin in the nuclear compartment), normalized to nuclear volume ($\sum Vol_{Nuclear}$) and calibrated with InSpeck™ Normalization Factor ($II_{Norm}$). Similarly, the relative concentration of β-catenin in the Cytosol-Membrane compartment ($Conc_{\beta\text{-}cat,Cyto\text{-}Memb}$), and

the total intensity of β-catenin in the cytosol-membrane compartment $(\sum I_{Cyto\text{-}Memb})$ were normalized to the Cytosol-Membrane volume $(\sum Vol_{Cyto\text{-}Memb})$ and calibrated with $II_{Norm}$. The relative β-catenin whole cell concentration ($Conc_{\beta\text{-}cat,\ Whole\ Cell}$) was the summation of $Conc_{\beta\text{-}cat,\ Nuclear}$ and $Conc_{\beta\text{-}cat,Cyto\text{-}Memb}$ .

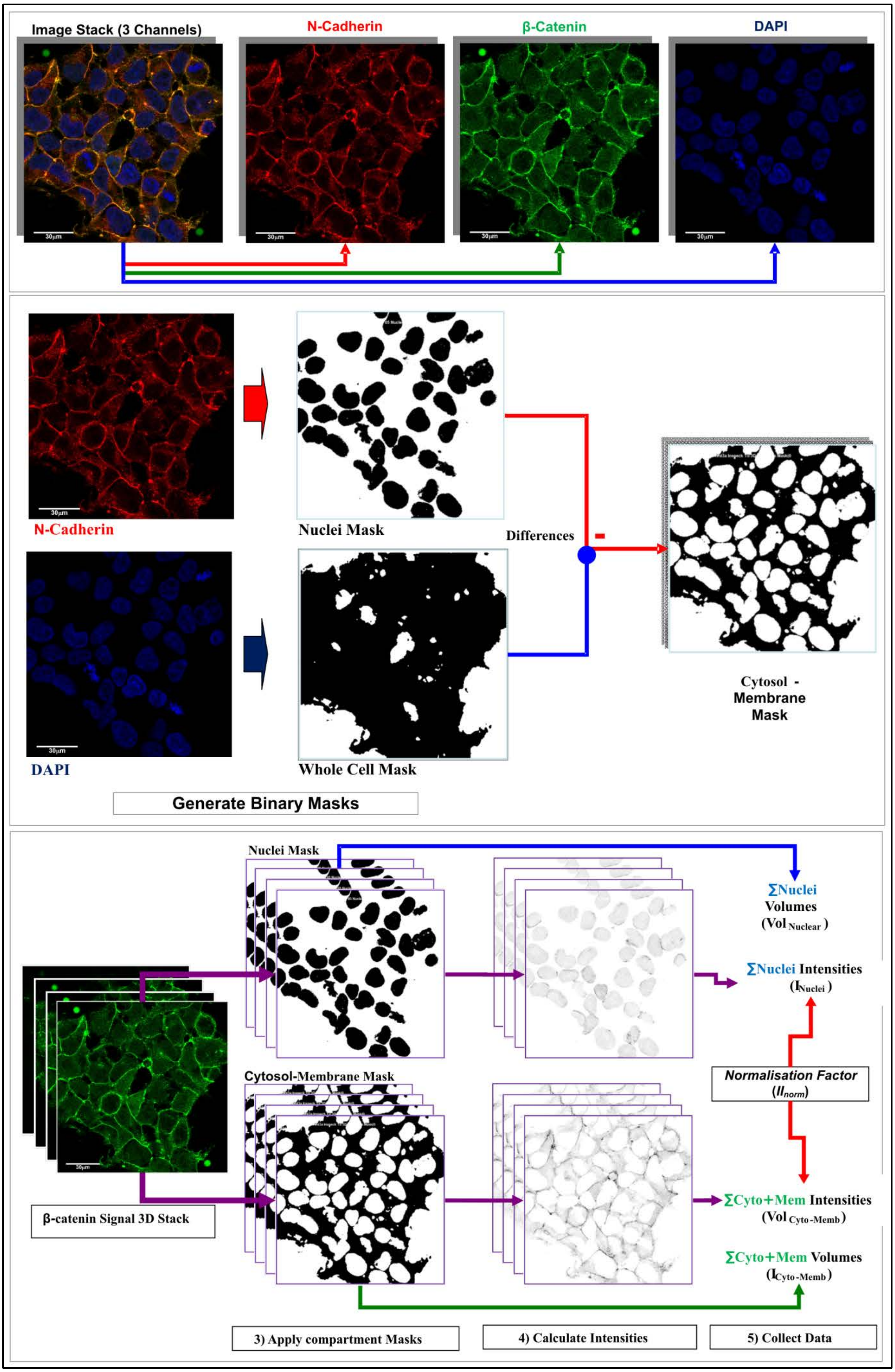

**Figure S5. 3D Compartmental Analysis of 3 fluorescence signals in HEK293T cells.**

The three fluorescence channel OIB image stacks was split into their three constituent signal channels representing staining for DAPI (blue) and immuno-staining for β-Catenin (green) and N-cadherin (red). The DAPI (blue) and N-cadherin (red) image intensities were selectively thresholded (defined level for object selection) and binarized to give the nuclei mask and whole cell mask respectively. The difference between the whole cell and nuclei mask generated the cytosol-membrane mask (binary subtraction). 3D compartmental masks were overlaid onto the β-catenin signal 3D stack to isolate the respective compartment β-catenin intensities so that the total intensities for nuclei ($\sum I_{Nuclear}$) and cyto-membrane ($\sum I_{Cyto+Memb}$) compartments could be calculated. The respective compartment volumes ($Vol_{Nuclear}$ and $Vol_{Cyto+Memb}$) were calculated from the masks. A normalization factor ($II_{norm}$) was used to allow the comparison of intensities between image sets. Scale Bars: 30µm.

### 1.5 Computational Modeling: Optimization Tools

***Sensitivity Analysis***:

With the large number of reactions and parameters in the model, the influence of each parameter on the system was different. A systematic investigation was conducted to identify the parameters which were most influential in the system. These parameters formed the target parameters to be used in optimization. This analysis identifies reactions with the highest influence on system behavior (in this case, free β-catenin concentration). 'Control coefficients' [4, 5] were defined (Equation S5) which measure relative changes in steady state in a particular variable (free β-catenin) with respect to changes in a specific parameter. Parameters were systematically scaled by factors of $e^{\pm n}$ where $n = 1, 2 \text{ or } 3$ for an adequate coverage of parameter space.

$$\text{Control Coefficient}: C_{\pm i}^{\beta-catenin} = \frac{K_{\pm i}}{\beta - catenin}\frac{\delta(\beta - catenin)}{\Delta k_{\pm i}} \qquad \textbf{Equation S5}$$

Where $k$ is the specific parameter being investigated and $i$ the scale factor applied ($i = e^{\pm n}$). For a broader representation of the sensitivity of the parameter, the analysis output δ(β-catenin) was calculated based on differences at 50 different time-points (t) from the steady state time-course either as a summation of differences $\sum_{1}^{50} \delta(\beta - catenin)$ $\sum_1^{50} \delta(\beta\text{-catenin})$, or summation of absolute differences $\sum_{1}^{50} abs[\delta(\beta - catenin)]$ $\sum_1^{50} \text{abs}[\delta(\beta\text{-catenin})]$ of the 50 different time-points or the difference at steady state $(\delta(\beta\text{-catenin}), t = 50)$. Importantly, there was no significant difference in results between these calculations, therefore the sum of absolute differences was employed in this study. The numbers of parameters to be adjusted for each analysis determined the number of permutations and complexity of the simulation. Here, three different permutation sets are defined and used, namely:

- Singular parameter change: adjusting one parameter at a time;
- Double parameter changes (with single scaling): simultaneously adjusting two parameters both scaled by the same factor; and
- Double parameter changes (with double scaling): adjusting two parameters at the same time but both parameters scaled by different factors;

Note: for the purpose of identifying specific sensitive parameters as targets for optimization, the method applied here was adapted and customized from the metabolic control analysis [4].

***Cost-minimization optimization:***

The optimization procedure used the Matlab function '*patternsearch*' routine in the '*Global Optimization Toolbox*' to search for optimal parameter values (for specified parameters) to minimize

the current model differences (cost) to the objective targets in order to obtained the closest possible model to the target experiment data. *Patternsearch* implements a direct search method for solving non-linear unconstrained optimization problems [6, 7].

***Steady State Analysis:***

The analyses for each model involved running the simulations until a steady state was achieved. These results allowed an overview of the protein distribution among the complexes at that particular steady state. The analysis provided static information on how the rates and model structure affected the distribution of key proteins. An assessment was then conducted on the distribution of complexes related to interactions, network structure and reaction rate within the model. This procedure was loosely termed 'Steady State Analysis'. The information obtained was used to inform the selection of parameters for adjustment during optimization; particularly, during topological analysis.

***Topology Analysis*:**

A Topological analysis is the logical process of relating the structure and interacting components of the model network to the required objective outcome of the optimization. Potential reactions which were critical for obtaining a specific outcome were identified and the direction (magnitudes) of adjustments assessed. Topological analysis allowed identification of structurally dependent reactions which affect steady state levels and transient behaviors and to logically estimated/approximate directions and magnitudes of adjustments.

### 1.6 Model 1 Optimized Equations and Parameters

Model 1 was constructed based on Lee *et al* model [8] structure (schematic as shown in Figure 6). Lee *et al*'s initial rates were applied and the mammalian protein concentrations from our previous work [1] were applied as initial total protein concentrations. The resulting optimized model parameters (Table S2), steady state protein concentrations (Table S3), kinetic rate equations (Table S4) and rate law governing rate of change (differential) for each species (Table S5) are as shown in the supplementary tables. The amount of fold changes that were applied during the optimization are shown in Table S6. Model 1 SBML and Matlab script files are available from the corresponding author upon request.

**Table S2. Optimized Parameters for Model 1.**

| *Parameters* | *Optimized values* | *Parameters* | *Optimized values* |
|---|---|---|---|
| K01 | 0.182 | K13 | 0.00010152 |
| K02 | 0.0182 | K14 | 3.0255 |
| K03 | 0.05 | K15 | 0.07515 |
| K04 | 0.267 | ka16 | 0.00234 |
| K05 | 0.133 | kd16 | 0.372 |
| ka06 | 0.0909 | ka17 | 0.0018 |
| kd06 | 0.909 | kd17 | 0.402 |
| ka07 | 1 | ka18 | 0.018 |
| kd07 | 1190 | kd18 | 0.378 |
| ka08 | 1 | ka19 | 0.0078 |
| kd08 | 7120.896 | kd19 | 1.3108 |
| K09 | 206 | ka20 | 0.00147 |
| K10 | 206 | kd20 | 0.231 |
| K11 | 0.417 | ka21 | 0.00084 |
| K12 | 1.3056 | kd21 | 0.10368 |

**Table S3. Initial and Steady State Concentrations (Optimized) for Model 1.**

| *Species* | *variable* | *Initial Concentrations (nM)* | *Optimized Steady State Concentrations (nM)* |
|---|---|---|---|
| DshI | x(1) | 100 | 100 |
| DshA | x(2) | 0 | 0 |
| Wnt | x(3) | 0 | 0 |
| APC/Axin/GSK3 | x(4) | 0 | 0.063 |
| APC/Axin | x(5) | 0 | 0.051 |
| GSK3 | x(6) | 70.9 | 12.3 |
| APCp/Axinp/GSK3 | x(7) | 0 | 0.13 |
| Bcat/APCp/Axinp/GSK3 | x(8) | 0 | 0.0062 |
| Bcat | x(9) | 494.7 | 349.9 |
| Bcatp/APCp/Axinp/GSK3 | x(10) | 0 | 0.0062 |
| Bcatp | x(11) | 1 | 3.05 |
| Axin | x(12) | 144.99 | 40.3 |
| APC | x(13) | 4.24 | 1.5 |
| Bcat/APC | x(14) | 0 | 2.4 |
| Bcat/TCF | x(15) | 0 | 10.3 |
| TCF | x(16) | 15 | 4.7 |
| GSK3/Axin | x(17) | 0 | 23.5 |
| Axin/Bcat | x(18) | 0 | 83.8 |
| GSK3/APC | x(19) | 0 | 0.12 |
| GSK3/BCat | x(20) | 0 | 34.8 |

**Table S4. Reaction rate equations and associated variables for Model 1.**

| *Reactions* | *Rate equations* |
|---|---|
| V01 | K01×(x(1))×(x(3)) |
| V02 | K02×(x(2)) |
| V03 | K03×(x(2))×(x(4)) |
| V04 | K04×(x(4)) |
| V05 | K05×(x(7)) |
| V06 | ka06×(x(5))×(x(6))-kd06×(x(4)) |
| V07 | scale1×(ka07×(x(12))×(x(13))-kd07×(x(5))) |
| V08 | scale2×(ka08×(x(9))×(x(7))-kd08×(x(8))) |
| V09 | K09×(x(8)) |
| V10 | K10×(x(10)) |
| V11 | K11×(x(11)) |
| V12 | bcat_production×K12 |
| V13 | K13×(x(9)) |
| V14 | axin_production×K14 |
| V15 | K15×(x(12)) |
| V16 | scale3×(ka16×(x(9))×(x(16))-kd16×(x(15))) |
| V17 | scale4×(ka17×(x(9))×(x(13))-kd17×(x(14))) |
| V18 | ka18×(x(12))×(x(6))-kd18×(x(17)) |
| V19 | ka19×(x(12))×(x(9))-kd19×(x(18)) |
| V20 | ka20×(x(6))×(x(13))-kd20×(x(19)) |
| V21 | ka21×(x(6))×(x(9))-kd21×(x(20)) |
| **Variables** | **Values** |
| scale1 | 2601.1254 |
| scale2 | 2600.75 |
| scale3 | 2599.9971 |

| | |
|---|---|
| scale4 | 2601.9683 |
| axin_production | 0 for CHX and 1 for SS and WNT3A |
| bcat_production | 0 for CHX and 1 for SS and WNT3A |

**Table S5. Rate of change equations for each species (Model 1).**

| *Species* | *Rate of change of species (dSpecies/dt)* |
|---|---|
| DshI | -V01+V02 |
| DshA | +V01-V02-V03 |
| Wnt | -V01 |
| APC/Axin/GSK3 | +V06-V04+V05-V03 |
| APC/Axin | -V06+V07+V03 |
| GSK3 | -V06+V03-V18-V20-V21 |
| APCp/Axinp/GSK3 | +V04-V05-V08+V10 |
| Bcat/APCp/Axinp/GSK3 | +V08-V09 |
| Bcat | -V08+V12-V13-V16-V17-V19-V21 |
| Bcatp/APCp/Axinp/GSK3 | +V09-V10 |
| Bcatp | +V10-V11 |
| Axin | -V07+V14-V15-V18-V19 |
| APC | -V07-V17-V20 |
| Bcat/APC | +V17 |
| Bcat/TCF | +V16 |
| TCF | -V16 |
| GSK3/Axin | +V18 |
| Axin/Bcat | +V19 |
| GSK3/APC | +V20 |
| GSK3/BCat | +V21 |

**Table S6. Selected parameters of the Model 1 which were adjusted in this study.**
The original Lee *at al.* (2003) and newly optimized/validated parameter values for Model 1. The last column depicts the degree of adjustment made to the parameters (with respect to the original value by Lee *et al.2003*) after the optimization of Model 1.

| *Parameter* | *Parameter values* | | *Δ fold changes from Lee et al.* |
|---|---|---|---|
| | Lee *et al* 2003 | Model 1 Optimized | |
| kd07 | 50 | 1190 | 24 |
| kd08 | 120 | 7121 | 59 |
| k12 | 0.42 | 1.31 | 3 |
| k13 | 0.000257 | 0.000102 | 0.395 |
| k14 | 0.0000822 | 3.0255 | 36807 |
| k15 | 0.167 | 0.07515 | 0.450 |

### 1.7 Numerical Analysis for Model 2 (Simplified Two Compartment β-catenin Model)

Initially, HEK293T cells had a total β-catenin concentration of 490±90 nM [1] and a compartment concentration ratio (non-Nuclear:Nuclear) of 0.95. This implied a β-catenin estimation of 516nM in the nuclear compartment and 466nM in the Non-Nuclear compartment. In the absence of synthesis, total β-catenin concentration in the cytosol-membrane and nucleus decreased at the same rate (Figure 4B). After 240 minutes, the concentration was ~5/6$^{th}$ of the initial condition (i.e. a 15-20% decline). After Wnt3A stimulation, total β-catenin concentration in the nucleus relative to the initial concentration increased faster than in the cytosol-membrane compartment relative to the initial

concentration (Figure 4B). The nucleus levels remain high even after 240 minutes.

Assumptions for the Two Compartment Model:

- There are two compartments, one for the cytosol-membrane and the other for the nucleus.
- β-catenin is only synthesized and degraded in the cytosol-membrane compartment.
- In each compartment there is a generic binding partner (ligand) for β-catenin.
- Synthesis and degradation of ligands is not included in the model.
- β-catenin can be exchanged between the compartments by diffusion.
- β-catenin can be actively transported between the compartments.
- The volumes of each compartment are taken from our earlier study [1].
- During cycloheximide inhibition experiments β-catenin synthesis is stopped; $B_{synthesis} = 0$.
- During Wnt stimulation experiments β-catenin degradation is stopped; $k_{degradation} = 0$

For the Topology and schematic diagrams of Model 2 see Figure 6.

**1.8 Estimation of initial conditions and parameter values**

The initial parameters for Model 2 are as shown in Table S7, some parameters have been estimated. The model parameters were then optimized against the experimental β-catenin steady state conditions and the time-courses under cycloheximide and Wnt. Some of the initial parameter values were taken from our previous work [1], namely the cellular compartment volumes and total β-catenin concentration in the two compartments of interest. As we were using a generic binding ligand for β-catenin, the equilibrium constants had to be chosen. We used a well-known binding partner of β-catenin, namely E-cadherin. Consequently, the initial equilibrium constant $k_D^C$ was estimated to be 260 nM. As for the nuclear equilibrium constant, the generic nuclear binding partner was assumed to be TCF and the initial constant used by Lee et al [8] (30nM) was used as the equilibrium constant $k_D^N$. The β-catenin synthesis rate was inferred from the optimized value obtained for Model 1 while the β-catenin degradation rate was assumed. Initial ligand concentrations (both in the CM and N) were assumed to be in excess, which we assumed to be 1000nM.

In this study, the active transport mechanism was again considered to be generic with no specific transporter specified. Therefore we assumed arbitrary values of 4.5 $m \cdot min^{-1}$ for active transport from CM to N and 2.0 $m \cdot min^{-1}$ for active transport between N to CM as initial estimates. The higher CM-N active transport rate was in consideration of our experimental observation of the influx of β-catenin into the nucleus at the initial onset of Wnt stimulation. Note: models were also developed with the active transport between compartments set to zero.

In terms of the intracellular compartment diffusion in Model 2 ($k_{diffusion}$), a particular characteristic of β-catenin was its ability to translocate into the nucleus without having any nuclear localization signal (NLS) sequence and independently of cellular nuclear import mechanisms. It was recently reported that β-catenin interacts directly with the nuclear pore complex (NPC) to facilitate its transport across the nuclear [9, 10] via the armadillo repeats. As no diffusion rate constant ($k_{diffusion}$) for HEK293T was available in the literature, it was estimated as follows:

Some physical estimations were obtained from ref [11].

"*The nuclear envelope of a typical mammalian cell contains 3000-4000 pore complexes.*" [11]

*"A quantitative analysis of such data suggests that the nuclear pore complex contains a pathway for free diffusion equivalent to a water-filled cylindrical channel about 9 nm in diameter and 15 nm long"* [11]

This suggested that NPC complexes effectively reduce the surface area for β-catenin diffusion.

Total Surface Area of NPC, $A_N = 3000 \times \pi \times r^2$ , where r: radius of NPC = 9÷2 = 4.5 nm

Total surface area of NPC, $A_N$ = 190755 nm

Nuclear Membrane thickness of NPC, H = 15nm

Nuclear volume, $V = 10^{-15}$m

Diffusion coefficient, $D = 50^{-12} m^2 s^{-1}$ [12]

$k_{diffusion} \sim D \times \frac{A_N}{H \times V} \sim 0.6\ m \cdot s^{-1} \sim 36\ m \cdot min^{-1}$

We used this as an estimate for the passive transport for the initial diffusion rate constant in this study.

**Table S7. Initial starting parameters for Models 2.**

| *Description** | *Parameter* | *Source* | *Initial conditions* |
|---|---|---|---|
| β-cat synthesis rate | $B_{synthesis}$ | Calculated from Figure 4A | 1.31 $nm \cdot min^{-1}$ |
| β-cat degradation rate | $k_{degradation}$ | Assumed. | 0. 01632 $min^{-1}$ |
| Free total β-cat concentration in CM | $B_{0T}^{c}$ | From [1] with Figure 4B, N:CM ratio | 465.5nM |
| Free total β-cat concentration in N | $B_{0T}^{N}$ | From [1] with Figure 4B, N:CM ratio | 515.8nM |
| Total ligand concentration in CM | $L_T^c$ | Assumed | 1000 nM |
| Total ligand concentration in N | $L_T^N$ | Assumed | 1000 nM |
| Average compartment (CM) volume per cell | $V^c$ | [1] | 1.16 pL |
| Average compartment (N) volume per cell | $V^N$ | [1] | 0.65 pL |
| Equilibrium constant in CM | $k_D^c$ | Inferred from [13], E-cadherin/β-catenin interactions | 260 nM |
| Equilibrium constant in N | $k_D^N$ | Inferred from[8], TCF /β-catenin interactions | 30 nM |
| Diffusion rate constant | $k_{diffusion}$ | Assumed | 36 $m \cdot min^{-1}$ |
| Active transport from CM | $k_{active}^{C}$ | Assumed | 0 or 4.5 $m \cdot min^{-1}$ |
| Active transport from N | $k_{active}^{N}$ | Assumed | 0 or 2.0 $m \cdot min^{-1}$ |

* CM: Cytosol-Membrane; N: Nuclear; β-cat: β-catenin

## 1.9 Model Equations and Analysis

*Notations:*

$k_R^X$ - Rate constant (reverse) in compartment *X* of β-catenin and ligand.

$k_F^X$ - Rate constant (forward) in compartment *X* of β-catenin and ligand.

B - β-catenin concentration

C - β-catenin:ligand complex /bound β-catenin concentration

L - Ligand concentration

Subscript *m* denotes variable in moles.

Subscript *0* denotes variable at steady state.

Subscript *T* denotes total concentration.

No Subscript denotes free concentration.

Superscript *X* denotes compartment where *X* can be C (cytosol-membrane) or N (nucleus).

The equations describing the dynamics of β-catenin in each compartment in terms of moles are:

$$\frac{dB_m^C}{dt} = B_{synthesis_m} - k_{degradation} B_m^c + \left(k_R^C C^c - k_F^C B^c \left(L_T^c - C^c\right)\right) V^c - k_{diffusion}\left(B^C - B^N\right) - k_{active}^C B^C + k_{active}^N B^N$$

**Equation S6**

$$\frac{dB_m^N}{dt} = \left(k_R^N C^N - k_F^N B^N (L_T^N - L^N)\right)V^N + k_{diffusion}\left(B^C - B^N\right) + k_{active}^C B^C - k_{active}^N B^N$$

**Equation S7**

In terms of concentration these equations become:

$$\frac{dB^C}{dt} = B_{synthesis} - k_{degradation}B^C + k_R^C C^C - k_F^C B^C L^C - \frac{k_{diffusion}\left(B^C - B^N\right) + k_{active}^C B^C - k_{active}^N B^N}{V^C}$$

**Equation S8**

$$\frac{dB^N}{dt} = k_R^N C^N - k_F^N B^N L^N + \frac{k_{diffusion}\left(B^C - B^N\right) + k_{active}^C B^C - k_{active}^N B^N}{V^N}$$

**Equation S9**

Where $K_D^C = \frac{k_R^C}{k_F^C}$.

The equations for the free ligands and complexes are

$$\frac{dL^c}{dt} = k_R^c C^c - k_F^c B^c \left(L_T^c - C^c\right)$$

**Equation S10**

$$\frac{dC^c}{dt} = -k_R^c C^c + k_F^c B^c \left(L_T^c - C^c\right)$$

**Equation S11**

$$\frac{dL^N}{dt} = k_R^N C^N - k_F^N B^N \left(L_T^N - C^N\right)$$

**Equation S12**

$$\frac{dC^N}{dt} = -k_R^N C^N + k_F^N B^N \left(L_T^N - C^N\right)$$

**Equation S13**

The total β-catenin concentration in the cytosol is

$$B_T^C = B^C + C^C$$

**Equation S14**

A similar relationship, as Equation S14, can be written for the total β-catenin concentration in the nucleus, $B_T^N$.

From experimental observation, at steady state,

$$B_{0T}^C = \alpha B_{0T}^N$$

**Equation S15**

it was observed that $\alpha = 0.95$ in the HEK293T cells.

The time derivative of the total β-catenin is

$$\frac{dB_T^c}{dt} = \frac{dB^c}{dt} + \frac{dC^C}{dt}$$

**Equation S16**

So that according to Equation S8, Equation S11 and Equation S16

$$\frac{dB_T^c}{dt} = B_{synthesis} - k_{degradation}B^c - \frac{k_{diffusion}\left(B^c - B^N\right) + k_{active}^C B^c - k_{active}^N B^N}{V^c}$$

**Equation S17**

Likewise the time derivative of the total β-catenin in the nucleus is

$$\frac{dB_T^N}{dt} = \frac{k_{diffusion}\left(B^c - B^N\right) + k_{active}^C B^c - k_{active}^N B^N}{V^N}$$

**Equation S18**

Note that the time-change of total β-catenin in the nucleus depends only on the flux across the nuclear membrane, such that the concentration of nuclear β-catenin is a proxy for this flux. Whereas in the cytosol-membrane compartment, the time rate of change of total β-catenin is the sum of the flux across the nuclear membrane and the rate of synthesis and degradation.

Note that

$$\frac{1}{B_{0T}^c}\frac{dB_T^c}{dt} = \frac{B_{synthesis} - k_{degradation}B^c}{B_{0T}^c} - \frac{V^N}{V^C}\frac{1}{\alpha B_{0T}^N}\frac{dB_T^N}{dt}$$

**Equation S19**

where the LHS is the relative rate of change of total β-catenin in the cytosol-membrane compartment (normalized to the initial steady concentration). This is the quantity plotted in the transient experiments of the HEK293T under either Wnt or CHX perturbation. Similarly the quantity $\frac{1}{B_{0T}^N}\frac{dB_T^N}{dt}$ appearing in the RHS is the relative rate of change of total β-catenin in the nucleus and was also plotted in the experiments.

In the experiments, there was a difference between the rate of change of total β-catenin in the cytosol-membrane and nuclear compartments after Wnt stimulation. Treatment with cycloheximide (CHX) did not change the β-catenin concentration significantly.

## Steady state, Initial conditions

Initially, assuming that steady state conditions are occurring, from Equation S8 it follows

$$B_0^c = \frac{B_{synthesis}}{k_{degradation}}$$

**Equation S20**

We also see from **Equation S18** that

$$k_{diffusion}\left(B_0^c - B_0^N\right) + k_{active}^C B_0^c - k_{active}^N B_0^N = 0$$

**Equation S21**

*i.e.* the net flux between the compartments is zero at steady state. In the absence of active transport this can only be satisfied if $B_0^c = B_0^N$, that is the free β-catenin is equally divided between compartments. However active transport enables a difference in free β-catenin to be maintained during a steady state. Whether or not the concentration of free β-catenin is higher in the nucleus or cytosol, at a steady state, depends on the active transporters and the affinity of the binding partners within each compartment. The expectation is that active transport is a negligible contribution at a steady state.

Now

$$B_{0T}^c = B_0^c + C_0^C$$

**Equation S22**

where from **Equation S11**

$$C_0^c = \frac{B_0^c L_T^c}{K_D^c + B_0^c}$$

**Equation S23**

such that

$$B_{0T}^c = B_0^c + \frac{B_0^c L_T^c}{K_D^c + B_0^c}$$

**Equation S24**

A similar expression can be found for the nucleus.

$$B_{0T}^N = B_0^N + \frac{B_0^N L_T^N}{K_D^N + B_0^N}$$

**Equation S25**

Substituting Equation S24 and Equation S25 into Equation S21 we obtain a quadratic for $B_0^c$ which can be solved for the positive root

$$B_0^c = \frac{-[K_D^c + L_T^c - B_{0T}^c] + \sqrt{[K_D^c + L_T^c - B_{0T}^c]^2 + 4K_D^c B_{0T}^c}}{2} = \frac{B_{synthesis}}{k_{degradation}}$$

**Equation S26**

Likewise in the nucleus

$$B_0^N = \frac{-[K_D^N + L_T^N - B_{0T}^N] + \sqrt{[K_D^N + L_T^N - B_{0T}^N]^2 + 4K_D^N B_{0T}^N}}{2}$$

**Equation S27**

If $B_0^c = B_0^N$ (i.e. no active transporters)

$$(\alpha - 1)B_{0T}^N = C_0^C - C_0^N$$

**Equation S28**

The LHS is <0 and so

$$C_0^C < C_0^N$$

**Equation S29**

That is, at a steady state, in the absence of active transport, the complex concentration is higher in the cytoplasm-membrane compartment than the nuclear compartment.

### 1.10 Model 2 Numerical optimization and results

Using Matlab, the model was formulated using Equation S8-S13 and optimized numerically using the experimental results listed below.

- Relative change in total β-catenin concentration under Wnt3A or CHX perturbation (Figure 4A);
- Relative change in β-catenin nuclear/non-nuclear compartment concentration ratio under Wnt3A or CHX perturbation (Figure 4C); and
- Relative change in β-catenin compartment concentration (normalized to $t_0$) under Wnt3A or CHX perturbation (Figure 4B).

The following initial conditions were applied (tabulated in Table S5).

Steady state analysis was applied to obtain the steady state concentrations tabulated in Table S6. Note that $B_0^c = B_0^N$ and $C_0^c < C_0^N$

Upon perturbation:

CHX inhibition, $B_{synthesis}$= 0, $K_{degradation}$ = 0.01632, and $k_{active}^C = k_{active}^N = 0$

Wnt3A stimulation, $B_{synthesis}$= 1.3056, $K_{degradation}$ = 0, $k_{active}^C$= 4.5 and $k_{active}^N$ = 2.0

It was assumed active transport was stimulated by Wnt3A signaling. Two test cases were applied, one with active transport (initial $k_{active}^C$=4.5 and $k_{active}^N$ = 2.0) and one without active transport ($k_{active}^C$ and $k_{active}^N$ = 0). In the optimization, the parameter rates for $kR_{\_cyto}$, $kF_{\_cyto}$, $kR_{\_nuclear}$, $kF_{\_nuclear}$, $k_{diffusion}$ , $k_{active}^C$ and $k_{active}^N$ were adjusted. The steady state concentration was calculated before the perturbation analysis was conducted. The goodness of fit was calculated based on the steady state fit as well as the three sets of experimental results mentioned above. Please note that in this model, the Wnt3A stimulation was given a greater priority (twice) during optimization, in comparison with the inhibition. This was in order to capture the distinct dynamics of Wnt activation in comparison to the slow and variable rate of decrease due to CHX inhibition.

Model 2 SBML and Matlab script files are available from the corresponding author upon request.

# References


1. Tan CW, Gardiner BS, Hirokawa Y, Layton MJ, Smith DW, Burgess AW: **Wnt Signalling Pathway Parameters for Mammalian Cells.** *PLoS ONE* 2012, **7:**e31882.
2. Mathworks: **MATLAB.** In *Book MATLAB* (Editor ed.^eds.), version. 7.4.0.287 (R2007a) edition. City: The Mathworks, Inc; 2007.
3. Metamorph: **Metamorph Premier.** In *Book Metamorph Premier* (Editor ed.^eds.), 7.6.3.0 edition. City: MDS Analytical Tech; 2009.
4. Heinrich R, Schuster S: *The regulation of cellular systems.* Chapman & Hall; 1996.
5. Kacser H, Burns JA: **The control of flux.** *Symposia of the Society for Experimental Biology* 1973, **27:**65-104.
6. Kolda TG, Lewis RM, Torczon V: **Optimization by direct search: New perspectives on some classical and modern methods.** *Society for Industrial and Applied Mathematics* 2003, **45:**385-482.
7. Torczon V: **On the Convergence of Pattern Search Algorithms.** *SIAM Journal on Optimization* 1997, **7:**1-25.
8. Lee E, Salic A, Kruger R, Heinrich R, Kirschner MW: **The Roles of APC and Axin Derived from Experimental and Theoretical Analysis of the Wnt Pathway.** *PLoS Biology* 2003, **1:**E10.
9. Fagotto F, Glück U, Gumbiner BM: **Nuclear localization signal-independent and importin/karyopherin-independent nuclear import of β-catenin.** *Current Biology* 1998, **8:**181-190.
10. Yokoya F, Imamoto N, Tachibana T, Yoneda Y: **β-Catenin Can Be Transported into the Nucleus in a Ran-unassisted Manner.** *Molecular Biology of the Cell* 1999, **10:**1119-1131.
11. Alberts B, Johnson A, Lewis J, Raff M, Roberts K, Walter P: **Intracellular Compartments and Protein Sorting.** In *Molecular Biology of the Cell, 4th edition.* New York: Garland Science; 2002
12. Kühn T, Ihalainen TO, Hyväluoma J, Dross N, Willman SF, Langowski J, Vihinen-Ranta M, Timonen J: **Protein Diffusion in Mammalian Cell Cytoplasm.** *PLoS ONE* 2011, **6:**e22962.
13. Catimel B, Layton M, Church N, Ross J, Condron M, Faux M, Simpson RJ, Burgess AW, Nice EC: **In situ phosphorylation of immobilized receptors on biosensor surfaces: Application to E-cadherin/β-catenin interactions.** *Analytical Biochemistry* 2006, **357:**277-288.
